# Single-cell sequencing reveals karyotype heterogeneity in murine and human malignancies

**DOI:** 10.1186/s13059-016-0971-7

**Published:** 2016-05-31

**Authors:** Bjorn Bakker, Aaron Taudt, Mirjam E. Belderbos, David Porubsky, Diana C. J. Spierings, Tristan V. de Jong, Nancy Halsema, Hinke G. Kazemier, Karina Hoekstra-Wakker, Allan Bradley, Eveline S. J. M. de Bont, Anke van den Berg, Victor Guryev, Peter M. Lansdorp, Maria Colomé-Tatché, Floris Foijer

**Affiliations:** European Research Institute for the Biology of Ageing, University of Groningen, University Medical Center Groningen, A. Deusinglaan 1, Groningen, 9713 AV The Netherlands; Wellcome Trust Sanger Institute, Wellcome Trust Genome Campus, Hinxton, CB10 1SA UK; Department of Paediatrics, University of Groningen, University Medical Center Groningen, A. Deusinglaan 1, Groningen, 9713 AV The Netherlands; Department of Pathology & Medical Biology, University of Groningen, University Medical Center Groningen, A. Deusinglaan 1, Groningen, 9713 AV The Netherlands; Terry Fox Laboratory, BC Cancer Agency, Vancouver, BC V5Z 1L3 Canada; Division of Hematology, Department of Medicine, University of British Columbia, Vancouver, BC V6T 1Z4 Canada; Institute of Computational Biology, Helmholtz Zentrum München, Ingolstädter Landstr. 1, Neuherberg, 85764 Germany

**Keywords:** Aneuploidy, Karyotype heterogeneity, Single-cell sequencing, Copy number detection, Lymphoma, Leukaemia

## Abstract

**Background:**

Chromosome instability leads to aneuploidy, a state in which cells have abnormal numbers of chromosomes, and is found in two out of three cancers. In a chromosomal instable p53 deficient mouse model with accelerated lymphomagenesis, we previously observed whole chromosome copy number changes affecting all lymphoma cells. This suggests that chromosome instability is somehow suppressed in the aneuploid lymphomas or that selection for frequently lost/gained chromosomes out-competes the CIN-imposed mis-segregation.

**Results:**

To distinguish between these explanations and to examine karyotype dynamics in chromosome instable lymphoma, we use a newly developed single-cell whole genome sequencing (scWGS) platform that provides a complete and unbiased overview of copy number variations (CNV) in individual cells. To analyse these scWGS data, we develop AneuFinder, which allows annotation of copy number changes in a fully automated fashion and quantification of CNV heterogeneity between cells. Single-cell sequencing and AneuFinder analysis reveals high levels of copy number heterogeneity in chromosome instability-driven murine T-cell lymphoma samples, indicating ongoing chromosome instability. Application of this technology to human B cell leukaemias reveals different levels of karyotype heterogeneity in these cancers.

**Conclusion:**

Our data show that even though aneuploid tumours select for particular and recurring chromosome combinations, single-cell analysis using AneuFinder reveals copy number heterogeneity. This suggests ongoing chromosome instability that other platforms fail to detect. As chromosome instability might drive tumour evolution, karyotype analysis using single-cell sequencing technology could become an essential tool for cancer treatment stratification.

**Electronic supplementary material:**

The online version of this article (doi:10.1186/s13059-016-0971-7) contains supplementary material, which is available to authorized users.

## Background

Chromosomal instability (CIN) is a process leading to structural and whole chromosome abnormalities and results in cells with an abnormal DNA content, a state defined as aneuploid. CIN and the resulting aneuploidy cause physiological stress and growth defects in yeast and primary mouse embryonic fibroblasts [1, 2]. Furthermore, some of the mouse models that were engineered to model CIN are characterised with a reduced lifespan, which can be rescued by reducing the levels of CIN [3–5]. Although aneuploidy has detrimental consequences for untransformed cells, more than two out of three cancers are aneuploid, suggesting a fundamental relationship between aneuploidy and tumourigenesis that so far remains poorly understood [6–8].

Various mouse models have been engineered to investigate the relationship between aneuploidy and tumourigenesis, typically by abrogating components of the spindle assembly checkpoint (SAC) [9–11]. The SAC monitors chromosome segregation by blocking mitotic cells in metaphase until proper kinetochore-microtubule attachment and tension have been established [12–14], and its (partial) abrogation will therefore lead to CIN. We have previously shown that CIN as provoked by truncating the SAC kinase Mps1 selectively in murine T-cells is not sufficient to trigger malignant proliferation. However, when combined with loss of p53, CIN significantly accelerated lymphomagenesis. When we analysed the average chromosome content in the emerging T cell acute lymphoblastic lymphomas (T-ALLs) by array comparative genomic hybridisation (aCGH), we found that all lymphomas displayed highly similar karyotypes, suggesting clonal selection for the recurring chromosomal abnormalities [15]. This was surprising because we also showed that Mps1 truncation results in mitotic abnormalities in virtually each cell division in tissue culture cells, which would counteract clonal selection of any chromosomal abnormality in a tumour. One possible explanation is that aneuploid T-ALL cells can overcome the CIN phenotype and thus maintain a stable karyotype. Alternatively, selection forces for specific chromosome alterations outcompete the mis-segregation events. The resulting chromosomal imbalances are expected to have severe effects on gene expression and thus cellular fitness. Although most forms of aneuploidy are expected to decrease fitness, karyotype heterogeneity could result in selection of cells that have accumulated favourable copy numbers of chromosomes expressing genes important for tumour evolution and overall cellular fitness.

Indeed, karyotype heterogeneity occurs in human cancers [16, 17], has been associated with human tumour evolution, and might therefore impact therapeutic response to cancer therapy [18]. However, most current cytogenetic and molecular techniques are limited in the number of karyotype alterations per cell they can detect, are biased towards dividing subpopulations, or can only measure the population-average chromosome copy number alterations [19, 20]. These shortcomings have precluded thorough analysis of intratumour chromosome copy number variations. Recent advances in single-cell genomics allow researchers to dissect the heterogeneity of the cancer genome with greater resolution than ever before [21, 22].

In this study we describe the application of single-cell whole genome sequencing (scWGS) to map and quantify karyotype heterogeneity in primary mouse lymphoma and human leukaemia samples. For this purpose, we have developed a new bioinformatics tool, AneuFinder, to identify chromosome copy numbers in scWGS data. Using these tools, we now show that besides recurrent chromosome copy number alterations, the aneuploid T-ALLs that arise in our mouse model display high-grade ongoing CIN as evidenced by severe intratumour karyotype heterogeneity. Importantly, analyses of a number of human paediatric B cell acute lymphoblastic leukaemia (B-ALL) samples using this platform revealed different grades of karyotype heterogeneity, demonstrating that CIN rates differ between these malignancies. Therefore, as ongoing CIN is an important hypothesised driver of cancer evolution [17], our platform might become an important tool to predict treatment outcome or even treatment stratification.

## Results

### Chromosomal instable T-ALLs show recurrent chromosome copy number changes

**Fig. 1:**
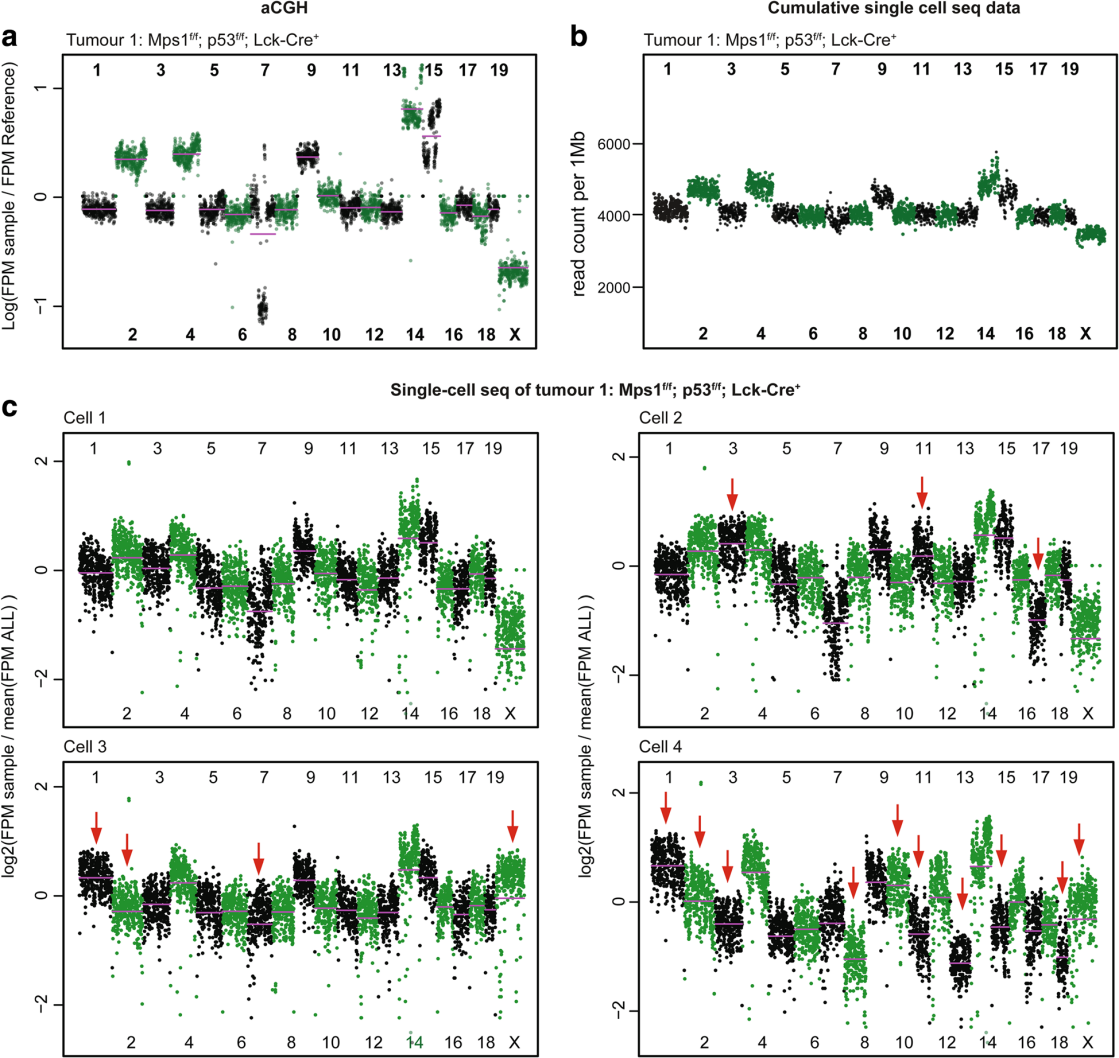
Chromosomal instable T-ALL display recurring chromosome copy numbers, as assessed by array CGH. **a** Two representative T-ALLs analysed using array CGH, compared to a euploid reference, showing recurrent gains of chromosomes 4, 9, 14 and 15, and other tumour-specific alterations. The *purple bars* indicate the mean log-value of the respective chromosome. **b** Cumulative single-cell sequencing libraries to simulate bulk data, showing a comparable karyotype as found by aCGH. **c** Single-cell sequencing analysis of four representative cells from T-ALL 1, showing identical chromosome copy numbers to the aCGH profile (*cell 1*), or cell-unique copy numbers (*cells 2, 3 and 4*; *red arrows*)

We previously reported that CIN in T-cells induced by a truncation of the SAC kinase *Mps1* synergises with *p53* loss in lymphomagenesis [15]. When we re-examined our earlier aCGH data [15], we again identified recurrent chromosome copy number changes in a large cohort of aneuploid lymphomas, most notably gains of chromosomes 4, 9, 14 and 15 (Fig. 1a, Additional file 1: Figure S1). The fact that these recurrent chromosomal abnormalities were detectable by bulk measurement aCGH (i.e. measuring the average copy number changes in a piece of tumour and therefore millions of cells) indicates that the majority of the T-ALL cells in the individual lymphomas displayed these aneuploidies [19]. Indeed, when we determined chromosome 15 aneuploidy in individual cells using interphase FISH, we confirmed that >70 % of the cells had three or more copies [15]. As *Mps1* truncation is expected to cause ongoing chromosome instability, these clonal karyotypes were unexpected. Two possible explanations for this are: (1) the malignancies somehow compensate for *Mps1* truncation, thus alleviating the CIN phenotype; or (2) the ongoing CIN is outcompeted by a selection that ultimately drives lymphoma cells to converge towards favourable chromosome-specific copy number states. If the latter explanation is true, T-ALLs should display cell-to-cell variability for chromosome numbers, i.e. karyotype heterogeneity [15].

Traditional methods to examine karyotypes depend on dividing cells (in case of normal and spectral karyotyping [SKY]), or are limited in the number of chromosomes that can be quantified per cell (in case of interphase FISH). An alternative to measure copy number alterations in a tumour is to measure the average DNA content (e.g. by aCGH) [19, 23], but this obscures intratumour heterogeneity. We therefore moved to single-cell sequencing as a method for karyotyping, making use of a modified scWGS protocol, described in more detail in van den Bos et al. [24]. Briefly, this scWGS platform involves single-cell sorting of primary tumour cells as nuclei by flow cytometry, followed by automated DNA fragmentation, barcoded next generation sequencing library preparation and shallow multiplexed sequencing [24]. To validate our platform, we first sequenced the genomes of 25 primary T-ALL cells isolated from an *Mps1*^*f/f*^*; p53*^*f/f*^*; Lck-Cre*^*+*^ lymphoma that we had previously assessed [15] by aCGH-analysis (T-ALL 1, aCGH data in Fig. 1a). We first compared the single-cell sequencing data to the existing aCGH data by creating an artificial ‘bulk sequencing file’ that has the cumulative data of all individual single-cell sequencing libraries (Fig. 1b) to determine how representative the sampled cells are for the bulk tumour. Indeed, we found that the copy number changes in the bulk sequencing analysis were identical to those observed in the aCGH data (compare Fig. 1a and b). However, when we plotted the individual single-cell libraries, we detected many additional copy number changes: while some of the single cells showed the exact karyotype as found by aCGH analysis (Ts2, Ts4, Del7, Ts9, Ts14, Ts15; Fig. 1c; cell 1, more examples in Additional file 2: Figure S2), most cells displayed additional chromosomal aberrations (Fig. 1c; compare cells 2, 3 and 4 to cell 1, more single-cell libraries in Additional file 2: Figure S2), reflecting karyotype heterogeneity and suggestive of ongoing CIN. Indeed, when we manually annotated the individual karyotypes of all 25 cells, we found that 56 % of the cells had a unique karyotype (Additional file 3: Figure S3), further emphasising the heterogeneity that our earlier aCGH analysis had failed to detect.

### AneuFinder: a tool to analyse high throughput single-cell sequencing data

**Fig. 2:**
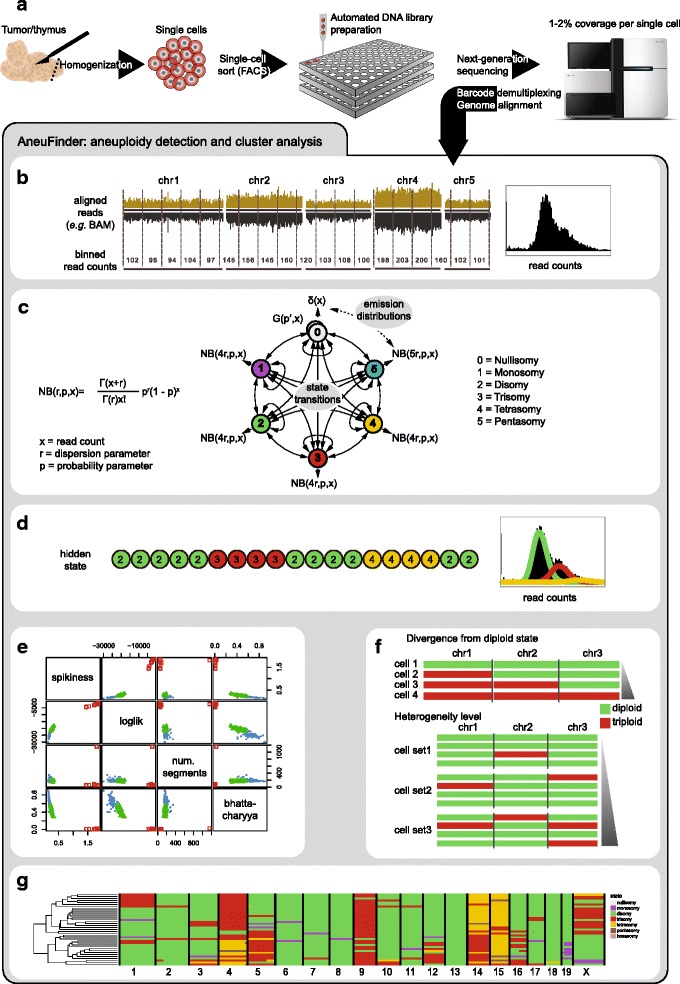
AneuFinder – automated copy number analysis of single-cell sequencing data. **a** Samples are homogenised, single-cell sorted and sequenced. **b** Aligned sequencing reads are counted in non-overlapping bins of variable size based on mappability. **c** A Hidden Markov Model with multiple hidden states is applied to the binned read counts in order to predict copy number state of every single bin. Emission distributions are modelled as negative binomial distributions (NB (r,p,x)). **d** The model parameters are estimated using the Baum Welch algorithm and every binned read count is assigned to the copy number state that maximises the posterior probability. **e** Quality of each single-cell library is assessed based on the following measures: spikiness, loglikelihood of the model determined by the Baum-Welch algorithm, number of separate copy number segments and Bhattacharyya distance. Libraries are clustered based on these measures: the highest scoring cluster is selected for further analysis. **f** The extent of aneuploidy is measured as the divergence of a given chromosome from the normal euploid state. At the cell population level, heterogeneity is measured as the number of cells with a distinct copy number profile within the population. **g** Example of a genome-wide copy number profile of a population of T-ALL cells. Each *row* represents a single cell with chromosomes plotted as *columns*. Copy number states are depicted in *different colours*. Cells are clustered based on the similarity of their copy number profile

While the scWGS data provided greater insight into the diversity of karyotypes than ‘bulk’ aCGH analysis, annotating the individual karyotypes is labour-intensive, and, more importantly error prone and possibly biased as karyotypes are annotated by visual inspection. Furthermore, a minority (~11 % of the libraries, Additional file 4: Table S1) of the single-cell sequencing libraries are of poor quality, making unbiased quality control essential for faithful copy number annotation. To automate quality control, and to facilitate copy number calling of single-cell sequencing data, we developed an automated analysis pipeline called AneuFinder with the following key features: (1) independence of an external reference for copy number analysis; (2) automated quantification of CNVs using a Hidden Markov model [25]; (3) stringent semi-automated quality control of individual sequencing libraries; and (4) generation of BED files for an external genome browser to zoom into small amplified/lost regions. After sample sorting and sequencing (Fig. 2a), AneuFinder analyses the aligned sequencing reads (e.g. BAM files). The reads are counted in non-overlapping bins of variable size based on mappability, averaging at 1 Mb in size, followed by a GC correction (Fig. 2b, Additional file 5: Supplementary Materials & Methods). A Hidden Markov model is then applied to the binned sequencing data, assuming several possible (i.e. biologically relevant) states, from nullisomy up to decasomy (10 copies). All states are modelled by a negative binomial distribution, except for the nullisomy that is modelled by a delta distribution (see Additional file 5: Supplementary Materials & Methods). The Baum-Welch algorithm [25] is used to obtain the best fit for the distribution parameters, transition probabilities and posterior probabilities (Fig. 2c). Subsequently, each bin is assigned the copy number state with the highest posterior probability (Fig. 2d). Since single-cell sequencing can be inherently noisy, we included a stringent quality control using a multivariate clustering approach of individual libraries based on several quality measures calculated by AneuFinder. These include, for example, the bin-to-bin variation in read count (spikiness), the number of contiguous stretches of bins with the same copy number state and the Bhattacharyya distance [26] between the negative binomial distributions (also see Additional file 5: Supplementary Materials & Methods). We then select the best scoring cluster for downstream analysis, resulting in ~89 % of libraries being used in downstream analyses (Fig. 2e, Additional files 4 and 5: Table S1 and Materials & Methods). To assess the extent of karyotypic heterogeneity in a set of cells, we developed a heterogeneity score, as well as an aneuploidy score (divergence from euploidy; Fig. 2f, Additional file 5: Supplementary Materials & Methods, Additional file 6: Table S2). Finally, the copy number states are plotted in a genome-wide fashion, with cells being clustered based on the similarity of their copy number profile. We extensively compared the AneuFinder pipeline to another tool used for identification of CNVs in single-cell sequencing data, Gingko [27], and found that copy number calls were in general concordant between both methods, although Gingko was less sensitive than AneuFinder for the detection of small CNVs, while being more robust to sequencing noise (see Additional file 5: Supplementary Materials & Methods, and Additional file 7: Figure S4). The AneuFinder software was implemented as R-package *AneuFinder* and is freely available through Bioconductor: http://bioconductor.org/packages/AneuFinder/.

### scWGS and AneuFinder reveal karyotype heterogeneity in murine CIN T-ALLs

**Fig. 3:**
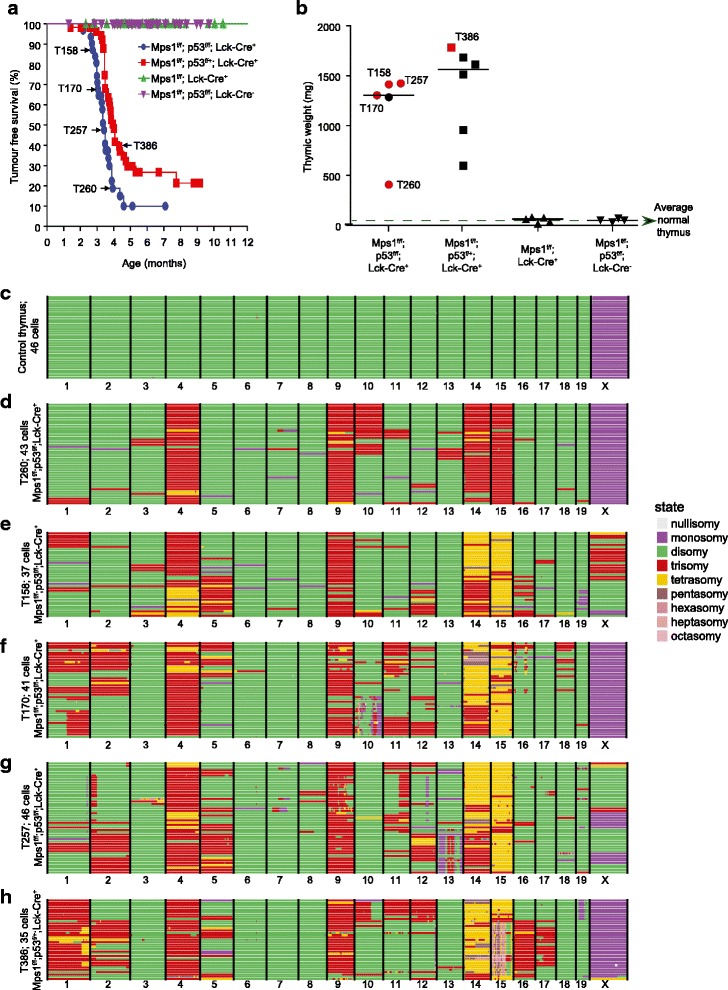
Single-cell sequencing analysis confirms both recurrent chromosome copy numbers and karyotype heterogeneity in CIN T-ALLs. **a** *Kaplan-Meier survival curve* of the listed genotypes. T-ALLs that were single-cell sequenced in this study are indicated with a *black arrow* and tumour ID. **b** Thymic/T-ALL weights of the listed genotypes. T-ALLs indicated in *red* were analysed using single-cell sequencing. **c**–**h** Genome-wide copy number plots as generated by the AneuFinder algorithm for six samples: one Lck-Cre^−^ control thymus and five CIN-driven T-ALLs. Individual cells are represented in *rows*, with the copy number state for ~1 Mb bins indicated in *colours* (see legend)

To further investigate karyotype heterogeneity in CIN lymphomas, we set up cohorts of *Mps1*^*f/f*^*; Lck-Cre*^*+*^, *Mps1*^*f/f*^*; p53*^*f/f*^*; Lck-Cre*^*+*^ and *Mps1*^*f/f*^*; p53*^*f/+*^*; Lck-Cre*^*+*^ mice, and *Lck-Cre*^*−*^ mice as controls. Mice were sacrificed when exhibiting signs of lymphoma (typically dyspnoea due to an enlarged thymus), thymuses were harvested and primary T-ALL single cell suspensions were frozen for subsequent single cell sequencing analysis. None of the *Mps1*^*f/f*^*Lck-Cre*^*+*^ (*n* = 71) or *Lck-Cre*^*−*^ animals (*n* = 63) succumbed to lymphoma within the first year (Fig. 3a, green and purple lines, respectively), while both the *Mps1*^*f/f*^*; p53*^*f/f*^*; Lck-Cre*^*+*^ (*n* = 31) and *Mps1*^*f/f*^*; p53*^*+/f*^*; Lck-Cre*^*+*^ (*n* = 58) developed T-ALLs with median latencies of 3.4 and 4.0 months, respectively (Fig. 3a, blue and red lines, respectively), identical as in our previous cohorts [15]. Thymic weights were in the range of 400–1420 mg (median: 1300 mg) and 590–1780 mg (median: 1560 mg) for *Mps1*^*f/f*^*p53*^*f/f*^*Lck-Cre*^*+*^ and *Mps1*^*f/f*^*p53*^*+/f*^*Lck-Cre*^*+*^ animals, respectively, while *Mps1*^*f/f*^*Lck-Cre*^*+*^ thymuses were 20–88 mg (median: 67 mg) and *Mps1*^*f/f*^*p53*^*f/f*^*Lck-Cre*^*−*^ control thymuses were 20–60 mg (median: 40 mg; Fig. 3b).

To determine aneuploidy at the single-cell level, we selected four *Mps1*^*f/f*^*; p53*^*f/f*^*; Lck-Cre*^*+*^ T-ALLs, one *Mps1*^*f/f*^*; p53*^*f/+*^*; Lck-Cre*^*+*^ T-ALL (Fig. 3a, arrows) and one control thymus (*Mps1*^*f/f*^*; p53*^*f/f*^*; Lck-Cre*^*−*^) for single-cell sequencing. For each sample, we sequenced 48 single-cell libraries. While AneuFinder did not detect any chromosome copy number alterations in the male Lck-Cre^−^ control thymus (Fig. 3c), all five T-ALL samples revealed clonal copy number gains of chromosomes 4, 9, 14 and 15 (Fig. 3d–h), highly similar as identified in *Mps1; p53*; *Lck-Cre* T-ALLs assessed by aCGH analysis (Fig. 1a and [15]). However, more importantly, AneuFinder also detected many other chromosome copy number variations that were present in a minority of the lymphoma cells that we predict aCGH analysis would have failed to detect. Indeed, these additional chromosome copy number alterations disappeared when we analysed our single - cell sequencing in bulk using the ‘bulk analysis sequencing files’ similar to Fig. 1b (Additional file 8: Figure S5). Furthermore, AneuFinder also revealed heterogeneity in the copy number states for the frequently gained chromosomes that we failed to detect previously by aCGH [15]. For instance, in tumours T158, T170 and T257 half of the cells had three copies of chromosome 14, while the other half had four or even more copies (compare Fig. 3e–g). All together, these data clearly show that *Mps1*^*f/f*^*; p53*^*f/f*^*; Lck-Cre*^*+*^ T-ALLs are not only highly aneuploid, but also display high-grade karyotype heterogeneity, suggestive of ongoing CIN.

### Quantifying karyotype heterogeneity in CIN T-ALLs

**Fig. 4:**
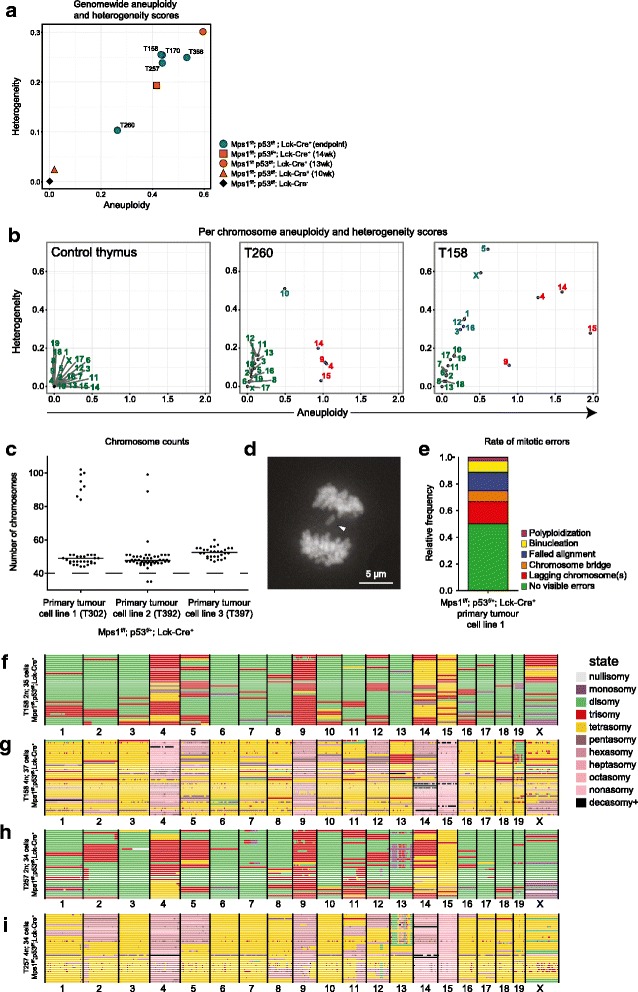
Early time point T-ALLs show similar levels of karyotype heterogeneity as endpoint lymphomas. **a** Aneuploidy and heterogeneity scores for the listed samples. The *black diamond* indicates the ‘baseline’ aneuploidy and heterogeneity based on the *Mps1* ^*f/f*^ *p53* ^*f/f*^ *Lck-Cre* ^*−*^ control thymus. **b** Aneuploidy and heterogeneity scores for a control thymus, T260, and T158 plotted per chromosome. Colours of the labels indicate clusters of chromosomes that favour a euploid copy number (*green*), show random copy number changes (*blue*) or favour copy number changes (*red*). **c** Chromosome counts acquired by metaphase spreads of three independently derived T-ALL cell lines (line 1, *n* = 35; line 2, *n* = 48; line 3, *n* = 30). Bars are median number of chromosomes (49, 47.5 and 51 for lines 1, 2 and 3, respectively). The *black dotted line* indicates the euploid chromosome count of 40 for mice. **d** *Still frame* of a mitotic cell from line 1 (T302) labelled with H2B-GFP, showing a lagging chromosome (*white arrowhead*). Frame is deconvolved and maximally projected. **e** Frequency of mitotic errors as analysed using live-cell time-lapse imaging of the H2B-GFP labelled lymphoma cell line 1 (*n* = 32). **f**, **g** Genome-wide copy number plots for G1 (**f**) cells and G2/near-tetraploid cells (**g**) for tumour T158. **h**, **i** Genome-wide copy number plots for G1 (**h**) cells and G2/near-tetraploid cells (**i**) for tumour T257

Our analyses so far revealed that the endpoint tumours in the *Mps1* mouse model are aneuploid with recurrent karyotypes for some chromosomes, but also display high-grade karyotype heterogeneity for most of the other chromosomes, suggesting ongoing CIN. To further quantify the extent of CIN (i.e. karyotype heterogeneity) during tumourigenesis, we next harvested *Mps1; p53; Lck-Cre* thymuses from mice at ages 10, 13 and 14 weeks when mice did not show any external evidence for T-ALL yet. Indeed, thymic weights were lower than endpoint lymphomas (40, 590 and 650 mg, respectively). While AneuFinder analysis revealed that few cells were aneuploid at 10 weeks, the karyotypes observed in the enlarged thymuses harvested from 13- and 14-week-old *Mps1; p53; Lck-Cre* mice were already very similar to those observed in endpoint T-ALLs (compare Additional file 9: Figure S6b, c to Fig. 3d–g) indicating that selection for the frequently gained chromosomes 4, 9, 14 and 15 occurs early in lymphoma development. We then compared the levels of karyotype heterogeneity between developing and endpoint T-ALLs. ‘Baseline’ heterogeneity in perfectly diploid Lck-Cre^−^ control T-cells was virtually zero (0.0009, Fig. 4a, black diamond). Indeed, the 10-week-old thymus showed an increase in the heterogeneity score (0.0226) compared to the control thymus as a result of copy number changes in the minority of the cells (Fig. 4a, karyotypes in Additional file 9: Figure S6a). Furthermore, five endpoint T-ALLs (Fig. 4a, blue circles, scWGS T-ALL karyotypes in Fig. 3d–g) showed a dramatic increase in the heterogeneity score (0.1032 – 0.2550), further emphasising the large variation in karyotypes present in those T-ALLs. Interestingly, the developing lymphomas harvested from 13- and 14-week-old *Mps1; p53; Lck-Cre* mice showed comparable heterogeneity and aneuploidy scores to the five assessed endpoint lymphomas (Fig. 4a, compare orange circle and square to blue circles, lymphoma karyotypes in Additional file 9: Figure S6b, c), suggesting that CIN rates remain constant during tumourigenesis in our mouse model.

Since different chromosomes showed varying degrees of gain or loss in the T-ALLs (Fig. 3d–h and Additional file 9: Figure S6b, c), we wondered whether calculating the aneuploidy and heterogeneity scores for individual chromosomes would reveal whether specific chromosomes more often showed changes in copy number than others. To this end, we plotted both scores per chromosome for all samples that were analysed by single-cell sequencing. For the control thymus, all chromosomes clustered together in the bottom left, indicating that none of the cells displayed chromosome copy number alterations (Fig. 4b, control thymus). In contrast, in the tumours we identified three ‘types’ of chromosomes: (1) chromosomes that were (virtually) never lost or gained (Fig. 4b, green chromosomes in T260 and T158), presumably due to lethality associated with such gain/loss events; (2) chromosomes that show a high heterogeneity rate, but low aneuploidy rate, for which copy number changes are presumably not selected for but that are tolerated relatively well (Fig 4b, blue chromosomes); and (3) chromosomes with high aneuploidy, but low(er) heterogeneity scores, for which the observed copy number changes are selected for in the tumours (Fig 4b, red chromosomes). These results (additional plots in Additional file 10: Figure S7) indicate that while some chromosomes copy number changes are favoured and others are not tolerated in these tumours, there is a third group of chromosomes for which copy number changes contribute to heterogeneity, but that are not (yet) selected for.

Even though we observed high levels of karyotype heterogeneity, the single-cell sequencing experiments are endpoint measurements and we therefore formally could not rule out that the T-ALLs consisted of a large number of different aneuploid but chromosomal stable clones. To determine whether chromosome mis-segregation events still occurred in the primary malignancies, we derived primary lymphoma cell lines from three endpoint T-ALLs. This yielded three viable primary murine T-ALL lines (T302, T392 and T397) that displayed chromosome numbers similar as observed in primary lymphomas as assessed by metaphase spread-based chromosome counting (median 47.5–51 for the T-ALL lines [passage 8], compared to 46–50 for the primary T-ALLs [compare Fig. 4c to Fig. 3d–h]). Furthermore, the lines displayed variability in chromosome numbers, indicating karyotype heterogeneity (Fig. 4c). We then labelled primary T-ALL cell line 1 (T302) with an H2B-GFP fusion protein to monitor the actual rates of chromosome mis-segregation by time-lapse microscopy. Indeed, we found that half of the mitoses (*n* = 32) showed clear mitotic abnormalities, mostly lagging chromosomes and failed alignments, similar to those observed previously in *Mps1*^*f/f*^*; p53*^*f/f*^ mouse embryonic fibroblasts (Fig. 4d, e, Additional files 11, 12 and 13: Movies 1–3 [15]). We therefore conclude that the karyotype heterogeneity that we observed in developing and endpoint *Mps1; p53; Lck-Cre* lymphomas is the result of ongoing CIN.

However, as we observed roughly 10 % of the mitoses in our primary T-ALL lines to result in tetraploid cells (Fig. 4d, e) and as for single-cell sequencing purposes we typically sort near diploid (2n) cells, we next investigated whether this resulted in an underestimation of the heterogeneity in endpoint tumours. To test this, we first examined the fate of T-ALL cells undergoing binucleation or polyploidisation events by time-lapse imaging in primary murine T-ALL cultures. While all cells displaying polyploidisation or binucleation events died between 4 and 11 h after mitotic exit (*n* = 7, e.g. Additional file 14: Movie 4), only 20 % of the cells that showed no clear mitotic abnormalities died in this experiment (*n* = 10). Even though this indicates that tetraploidisation events are selected against in a tissue culture setting, this still could be different in vivo, given that whole genome duplication is known to occur in human cancers. However, as with our existing strains we could not distinguish G2 cells from G1 tetraploid cells by FACS, we therefore analysed aneuploid murine T-ALLs for cells with a DNA content larger than 4n as an indication for proliferating tetraploid cells. Indeed, two out of the four analysed tumours (T158 and T257) had cells with a DNA content larger than 4n (see arrows Additional file 15: Figure S8a) indicating that some of the cells with a 4n DNA content had to be tetraploid. We then sorted individual G1 and G2-diploid/G1-(near-)tetraploid cells followed by single-cell sequencing from T158 (Fig. 4f, g) and T257 (Fig. 4h, i), respectively. Even though in this experiment we could not distinguish G2 cells from (near) tetraploid cells, the data revealed that near tetraploid cells were present as some of the cells had odd copy numbers of chromosomes and G2 cells by definition must have an even number of chromosome copies. Importantly, this allowed us to confirm that AneuFinder can detect such events: when we forced AneuFinder to fit the 4n population as diploid, the resulting fit was poor for cells with odd numbers (and thus true near-tetraploid cells; compare left and right hand panels in Additional file 15: Figure S8b, c) indicating that AneuFinder can detect tetraploid events even without *a priori* knowledge about the most prevalent state (diploid/tetraploid), similar to Ginkgo.

Further analysis of the 4n population of T158 revealed that 12 out of 37 analysed cells had an odd copy number for at least one of the chromosomes, indicating that at least 32 % of the 4n cells were true near-tetraploid and not G2 cells. The heterogeneity score for these near-tetraploid cells (0.6503) was much larger than for the 2n population (0.3099), indicating that the near-tetraploid cells can generate greater karyotype diversity than near-diploid cells, presumably because of the larger number of copy number states available. For the other 4n cells (68 %) we could not discriminate whether these were G2 cells or G1 near-tetraploid cells in this experimental setup. However, the 4n population only represented 4.1 % of all the live tumour cells, and therefore only between 1.3 % (32 % of 4.1 % near-tetraploid cells with odd numbers of chromosomes) and 4.1 % (all cells with near 4n DNA content) in tumour T158 were near-tetraploid cells. The contribution of these cells to intratumour heterogeneity is therefore limited and in agreement with the observed cell death events in the time-lapse experiments in primary aneuploid T-ALL cultures (Additional file 14: Movie 4). In the T257 4n population (5.3 % of all tumour cells) we found two out of 34 analysed cells to have odd chromosome numbers, representing ~0.3 % of the total tumour. Therefore, the contribution of genuine near-tetraploid cells to tumour T257 lies between 0.3 % and 5.3 %. We conclude that while the near-tetraploid cells contribute to intratumour heterogeneity, this contribution is limited, presumably due to cell death events after polyploidisation leading to low fractions of near-tetraploid cells in the primary aneuploid T-ALLs.

### Karyotype heterogeneity in human B cell acute lymphoblastic leukaemia

**Fig. 5:**
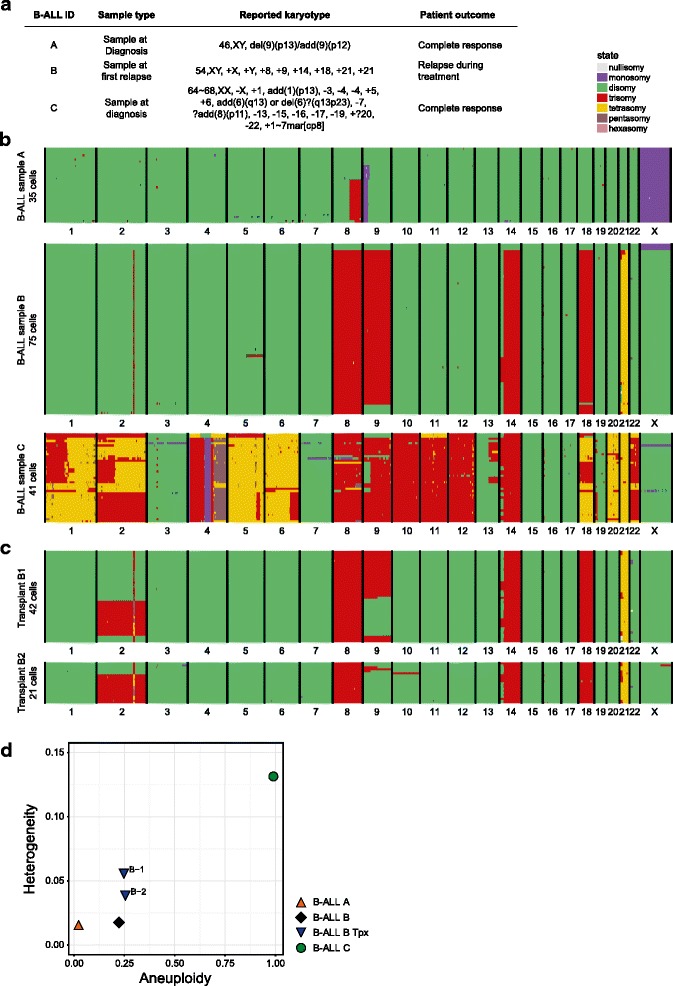
Karyotype heterogeneity of human B-ALLs increases upon engraftment into recipient mice. **a** Overview of B-ALL patient material used in this study. **b** Genome-wide copy number plots using ~1 Mb bins for bone marrow cells of three B-ALL patients. For B-ALL A and B, respectively, 8 and 3 euploid cells are present (non-cancer cells). **c** Genome-wide copy number plots using ~1 Mb bins for bone marrow cells of two mice, 28 weeks after engraftment with B-ALL B. **d** Aneuploidy and heterogeneity scores for the analysed B-ALL patient material and engraftments B-1 and B-2. The *orange triangle* indicates the baseline level of aneuploidy and heterogeneity of the near-euploid B-ALL A

Our results so far indicate that the lymphomas that arise in our *Mps1; p53; Lck-Cre* T-ALL model are aneuploid, with recurring chromosomes affected, and that chromosome numbers vary between cells for all chromosomes, resulting in intratumour karyotype heterogeneity. To investigate the relevance of our findings for human cancer, we next assessed to what extent human aneuploid tumours display karyotype heterogeneity. For this, we selected three B-ALL samples, one near-euploid (sample A), one intermediate aneuploid (sample B) and one highly aneuploid (sample C) as quantified by ‘traditional’ cytogenetics at the time of diagnosis (Fig. 5a). Indeed, when we analysed all single-cell sequencing libraries per tumour as bulk, we found the average karyotypes of the tumours to be similar to the reported cytogenetic reports (compare Fig. 5a to Additional file 16: Figure S9a). When assessed at the single-cell level, the near-euploid B-ALL A (Fig. 5b, top panel) did not show any whole chromosome copy number alterations, except for a deletion of 9p from p12 and p13 towards the telomeres. Interestingly, scWGS analysis also revealed a previously unidentified amplification on chromosome 8 in 20 out of 35 examined cells. Single-cell sequencing analysis of the intermediate aneuploid B-ALL B revealed that while the bulk of the cells had the karyotype as determined by traditional cytogenetic assessment, a small number of cells had a deviating karyotype. For instance, four cells lacked the extra copy of chromosome 9, and one cell did not have an extra copy of chromosome 18 (Fig. 5b, middle panel: B-ALL B). In addition, we identified a tumour-specific ~7.5 Mb CNV on chromosome 2 harbouring genes as *ITGA4*, *CERKL*, *PPP1R1C* and *PDE1A* (Additional file 16: Figure S9b), underscoring the potential of our platform for identifying structural abnormalities. Finally, the highly aneuploid B-ALL C showed a near-triploid karyotype with numerous whole chromosome and local copy number alterations and aneuploidy rates similar as observed in our *Mps1; p53; Lck-Cre* mouse model (Fig. 5b, bottom panel: B-ALL C). Indeed, when we quantified the karyotype heterogeneity using AneuFinder, we found that, while heterogeneity scores were relatively low for B-ALL A and B (0.0147 and 0.0176, respectively), heterogeneity in B-ALL C (0.1314) was nearing the heterogeneity scores observed in our aneuploid murine T-ALL samples (ranging: 0.1032–0.3010, compare Fig. 5d to Fig. 4a), emphasising the physiological relevance of our mouse model.

To assess whether the observed karyotype heterogeneity in the primary human B-ALL samples could be indicative for ongoing chromosomal instability, we next engrafted B-ALL B into sub-lethally irradiated immune-deficient mice. Mice were sampled every four weeks to assess the levels of chimerism (the fraction of human-specific CD45^+^ cells in the peripheral blood; Additional file 17: Figure S10), and were sacrificed when these levels became larger than 90 %. Interestingly, when we analysed the bone marrow of these mice (B-ALL B-1 and B-2) using scWGS and AneuFinder, we found that leukaemic cell karyotypes had changed after transplantation. In both transplanted samples, a fraction of cells gained chromosome 2 while none of the primary B-ALL B cells displayed chromosome 2 trisomy, and one cell in transplant B2 showed unique gain of chromosome 10 (Fig. 5c). In addition, the cells that had gained chromosome 2 had lost the extra copy of chromosome 9. This could be the result of a copy neutral loss of heterozygosity (cnLOH) event. To address this, we extracted all single nucleotide polymorphisms (SNPs) on chromosome 9 that were identified by our single-cell sequencing analysis in the cells harbouring two copies of chromosome 9. Unfortunately, we found that the coverage of the single-cell libraries was too low to sufficiently sample alternative alleles at defined SNP positions. To detect such events deeper sequencing is required (which increases cost price).

Heterogeneity scores for both transplants revealed an increased heterogeneity score for both transplanted leukaemias B1 and B2 (Fig. 5d, an increase from 0.0176 [black diamond] to 0.0565 and 0.0398 [blue triangles], respectively). Even though we formally cannot rule out that the cells with ‘new’ karyotype patterns were pre-existing in the primary leukaemia as low-abundant clones, these data strongly suggest that human tumours also exhibit ongoing CIN that can facilitate tumour evolution when tumour cells are exposed to stress, in this case survival and propagation in another niche (i.e. the mouse hematopoietic system).

## Discussion and conclusions

Aneuploidy is a hallmark of cancer cells and results from chromosome missegregation events during mitosis, a process called chromosomal instability (CIN). Indeed, most cancer cell lines exhibit CIN with ~10 % to 60 % of the cells displaying lagging chromosomes in mitosis, resulting in a distribution of cells with various aneuploidies [28–30]. However, while measuring CIN rates and the resulting karyotype variation in cancer cell lines is straight-forward, assessing karyotype heterogeneity in primary tumours is technically challenging [19]. Only recently, the first platforms to measure full karyotypes of single non-dividing primary cells have been reported [22, 23, 31].

In this study we describe a whole genome single-cell sequencing pipeline to quantify aneuploidy in primary (tumour) cells in an unbiased and high throughput fashion. We use this pipeline to measure karyotype heterogeneity in aneuploid murine T-ALLs that arise in our earlier published *Mps1; p53; Lck-Cre* mouse model as a measure for ongoing CIN [15]. We showed that our pipeline confirms our earlier observations that *Mps1; p53; Lck-Cre* lymphomas display recurrent chromosome copy number alterations for some chromosomes. In addition, we observed groups of chromosomes to show distinct degrees of aneuploidy and heterogeneity, with specific chromosomes favouring either aneuploidy or euploidy, and a third group of chromosomes showing apparent random copy number changes. This latter group of chromosomes could be important for tumour evolution as CIN events for these chromosomes are not selected against in the primary tumours. However, these copy number changes might become beneficial when the tumour environment changes, for instance during therapy and metastasis and thus lead to tumour recurrence or disease progression. It will therefore be extremely interesting to measure chromosome dynamics in primary tumours, metastases and recurring tumours in future experiments.

Using AneuFinder, we were able to detect high-grade karyotype heterogeneity that would likely have been obscured using other platforms such as array CGH. This is important for two reasons: (1) it confirms that chromosome mis-segregation is ongoing in the T-ALLs that arise in our *Mps1; p53; Lck-Cre* mice despite clonal selection for some chromosome number alterations and therefore that the selection forces outcompete mis-segregation rates. Indeed, we confirm ongoing CIN by showing that 50 % of the primary T-ALL cell divisions display mitotic abnormalities; and (2) it shows that karyotype heterogeneity as a result from high grade CIN will go unnoticed when using one of the most commonly employed platforms to assess (aCGH). Therefore, karyotype heterogeneity might be a gravely underestimated feature of cancer. Indeed, when we assessed karyotype heterogeneity in three paediatric B-ALL samples we found that heterogeneity greatly varied between the three assessed samples. The heterogeneity was not directly predictive of tumour outcome, as the two tumours with the lowest and highest heterogeneity rate had a favourable outcome while the tumour with medium CIN rates had a poor outcome. However, even though our sample size is too small to draw any conclusions, it is tempting to hypothesise that tumours benefit most from medium CIN rates as low CIN rates do not allow for any karyotype evolution and too high CIN rates prevent clonal selection of chromosomes when the environment has changed. This hypothesis appears to hold true in the mouse [32], but further studies comparing human tumours with known outcome are required to test this. Importantly, when we xenografted the human aneuploid B-ALL with a medium CIN rate into mice, we found that its propagation led to additional karyotypic changes, presumably the result of propagation in another niche in line with our hypothesis. Therefore, we argue that ongoing CIN and the resulting karyotypic heterogeneity are important factors to consider when predicting disease progression/outcome and potentially treatment response.

Genomic heterogeneity is a common feature of many cancers, but because of technical limitations, it is rarely properly quantified. Instead, primary tumour cell population averages are used for research and diagnostics purposes that may not entirely represent the diversity in the primary tumour [33]. The use of single-cell sequencing technology allows us to better estimate this level of tumour heterogeneity [22, 33, 34]. High resolution quantification of this heterogeneity is of the utmost importance as karyotype heterogeneity will drive tumour evolution and ultimately affect response to treatment [34–37]. While the mutational landscape of many tumour types has been characterised in detail, we have only begun to understand the role of karyotype heterogeneity in tumour evolution [18, 33, 38], a role that can be further examined using the scWGS-AneuFinder pipeline.

## Methods

### Animal housing and experiments

Animals used in this study had mixed C57BL/6 genetic backgrounds. All mice were bred in the Central Animal Facility (University Medical Centre Groningen [UMCG], Groningen, The Netherlands). The conditional deletion of *Mps1* was described before [15]. For survival studies, mice were monitored for tumour development starting at the age of 2.5 months by looking for signs of dyspnoea (a consequence of thymic hypertrophy) and general animal wellbeing.

### Ethics

Animal protocols were approved by the UMCG Committee on Animal Care (DEC) (RUG-DEC-6369). Human samples (bone marrow isolated from patients with acute lymphoblastic leukaemia) were collected as part of routine diagnostic procedures and leftover cells were used in this study (METc 2013.281). The use of these leftover samples was approved by the Medical Ethical Committee of the University Medical Center Groningen when patients (or guardians) had signed an informed consent letter. All patients and their guardians provided this written informed consent. All experimental methods comply with the Helsinki declaration for medical research involving human subjects.

### Array CGH data analysis

For the aCGH data analysis, probe signals were divided by corresponding signals of a euploid control reference and log_2_-transformed. The data were binned into 1 Mb segments in steps of 500 Kb and plotted. Array CGH data have previously been deposited in the National Center for Biotechnology Information Gene Expression Omnibus (NCBI GEO) database under accession no. GSE57334 [15].

### Single-cell whole genome sequencing and data processing

Single cells for the purpose of single-cell sequencing were isolated from thymuses by dissecting the organ and mincing the tissue through a 100 μm cell strainer. Cells were washed in PBS twice before stored in FBS with 10 % DMSO at −80 °C until sorted. Prior to sorting, cells were thawed, and incubated for 10 min on ice in the dark in a DNA-staining buffer containing 10 μg/mL propidium iodide and 10 μg/mL Hoechst, as well as 0.1 % Nonidet P-40 to dissociate the cytoplasm. The G1 population was sorted as individual nuclei into a 96-well plate format. Per sample, 10 nuclei were sorted into a single well to serve as a positive control, as well as an empty well acting as a negative control. Library preparation was performed using a modified scWGS protocol [24, 31] using a Bravo Automated Liquid Handling Platform (Agilent Technologies, Santa Clara, CA, USA). Clusters for sequencing were generated on the cBot (Illumina). Single-end 50 bp sequencing was performed on an Illumina HiSeq 2500 at ERIBA (Illumina, San Diego, CA, USA). Raw sequencing data were demultiplexed based on library-specific barcodes and converted to fastq format using standard Illumina software (bcl2fastq version 1.8.4). The resulting reads were mapped to mouse (mm10) or human (hg19) reference genome using Bowtie2 (version 2.2.4). Duplicate reads were marked using BamUtil (version 1.0.3). All single-cell sequencing data have been deposited at ArrayExpress under accession no. E-MTAB-4183.

### Copy number analysis using AneuFinder

Analysis of single-cell sequencing data was performed with the AneuFinder pipeline, using a blacklist strategy to exclude reads from artefact-prone regions. For analyses, we divided genomes into non-overlapping bins of variable size based on mappability with a mean of 1 Mb (for more details, see Additional file 5: Methods). Quality control was performed using a clustering approach (minimum of 1 and maximum of 3 clusters) based on the quality parameters described in the main text.

### T-ALL cell culture and live-cell time-lapse imaging

Mouse T-ALL cell lines were derived from primary mouse lymphomas and cultured as described by Jinadasa et al. [39]. Cells were incubated at 37 °C, 5 % CO_2_ and low oxygen (3 % O_2_). Cells were retrovirally transduced with a constitutive H2B-GFP construct (pSF91.2 H2B-GFP) by spinfection for 90 min at 1000 × *g* and 32 °C in retroviral supernatant containing polybrene 4 μg/mL (Sigma). Transduced cells were harvested, washed and seeded onto Glass Bottom Dishes (Greiner) 1 h prior to imaging. Mitoses were captured at 2-min intervals over the course of 16 h on a DeltaVision microscope (GE healthcare) at 100× magnification using SoftWorx software (GE Healthcare).

### In vivo leukaemia transplantation

Diagnostic bone marrow cells of patients with acute lymphoblastic leukaemia were thawed and transplanted by tail vein injection into sub-lethally irradiated immune deficient NOD/SCID/IL2Ry^−/−^ (NSG) mice, at a dose of 2.5 × 10^6^ cells/mouse. At 4-weekly intervals, blood was drawn from the retro-orbital plexus and leukaemia development was followed by flow cytometric analysis using the following markers against human antigens: CD19-PE, CD45-PECy7, CD34-APC, CD10-BV410 and CD20-BV605. Propidium Iodide was used to exclude dead cells. Human chimerism was defined as >1 % hCD45+ cells among total peripheral blood mononuclear cells (PBMC).

## Additional files

Additional file 1: Figure S1. Additional comparisons of aCGH and exome-sequencing analyses of T-ALLs driven by Mps1 and p53 mutation. Six additional T-ALLs analysed using array CGH, compared to a euploid reference, showing recurrent gains of predominantly chromosomes 4, 9, 14 and 15, and other lymphoma-specific alterations. (PDF 23139 kb)

Additional file 2: Figure S2. Additional single-cell sequencing analyses of Mps1 T-ALL 1. Single-cell sequencing plots for Mps1 T-ALL cells 1 (continuation of Fig. 1c). (PDF 63616 kb)

Additional file 3: Figure S3. Quantification of single-cell sequencing data of Mps1 T-ALL 1. **a** Quantification of the gains and losses of chromosomes for 25 Mps1 T-ALL 1 cells as analysed using single-cell sequencing (Additional file 2: Figure S2). Losses and gains were scored as 1 s and 3 s, respectively; 2 s indicates no change or disomies. The focal loss on chromosomes 7 was scored as a loss to discriminate cells that did not show this CNV. Fourteen out of 25 cells (56 %) displayed a unique karyotype. Cells with identical karyotypes are clustered together, resulting in 18 groups. **b** Frequency percentages of the gain, no change and loss events for chromosomes of Mps1 T-ALL 1. Gain of chromosome 4, 9, 14 and 15 are the most frequent events, occurring in >90 % of the cells, with gain of chromosomes 2 and the focal loss on chromosome 7 occurring in ~50 % of the cells. (PDF 548 kb)

Additional file 4: Table S1. Overview of single-cell sequencing samples and sequencing statistics. An overview of general sequencing and analysis statistics of the single-cell sequencing data. The number of analysed reads corresponds to the number of uniquely mappable reads used in the copy number annotation pipeline. (XLSX 38 kb)

Additional file 5: Supplementary Materials and Methods. (DOCX 35 kb)

Additional file 6: Table S2. Simulating the effects of different aneuploidies on the aneuploidy and heterogeneity score. Table showing the effect of modelling various aneuploidies on the aneuploidy and heterogeneity scores. (XLSX 42 kb)

Additional file 7: Figure S4. Examples of discordant copy number calls between AneuFinder and Ginkgo. *Top panels* show the AneuFinder profiles, *bottom panels* show the Ginkgo profiles, respectively. **a** Low quality library showing a highly segmented fit with AneuFinder. **b** Wrongly chosen ploidy state with Ginkgo. **c** *Red boxes* indicate chromosomes with unusually high read count dispersion where AneuFinder fails to assign a clear copy number state. **d** Small copy number change that is detected with AneuFinder but not with Ginkgo. (PDF 2236 kb)

Additional file 8: Figure S5. Cumulative single-cell sequencing data of control thymus and aneuploid T-ALLs. Copy number plots showing the reads per 1 Mb of cumulative single-cell sequencing data analysed as simulated bulk data, showing an obscuring effect on the karyotype heterogeneity. (PDF 3267 kb)

Additional file 9: Figure S6. Single-cell sequencing of early time point T-ALLs. Genome-wide copy number plots using ~1 Mb bins for three thymuses harvested from 10-, 13- and 14-week-old mice, showing high levels of karyotype heterogeneity at 13 and 14 weeks. (PDF 451 kb)

Additional file 10: Figure S7. Aneuploidy and heterogeneity per chromosome observed in a control thymus and T-ALLs. Aneuploidy and heterogeneity scores plotted per chromosomes of all T-ALLs examined in the study. Chromosomes indicated in *green* do not favour copy number change and show minimal heterogeneity. Chromosomes in *blue* show apparent random copy number changes. *Red* chromosomes favour copy number changes. (PDF 440 kb)

Additional file 11: *Time-lapse imaging* of a T cell labelled with H2B-GFP, showing a lagging chromosome. (MOV 3783 kb)

Additional file 12: *Time-lapse imaging* of a T cell labelled with H2B-GFP, showing tetraploidisation. (MOV 5675 kb)

Additional file 13: *Time-lapse imaging* of a T cell labelled with H2B-GFP, showing failed alignment. (MOV 2525 kb)

Additional file 14: *Time-lapse imaging* of a T cell labelled with H2B-GFP, showing tetraploidisation followed by cell death. (MOV 20714 kb)

Additional file 15: Figure S8. Single-cell sequencing of (near)-4n cells in T158 and T257. **a** PI/Hoechst FACS plots showing for four tumours, showing apparent cycling tetraploid cells in T158 and T257. **b** Comparison of AneuFinder copy number calling of T158; comparing the fit when forcing AneuFinder to call the majority state tetrasomy (*left*) or disomy (*right*). **c** Comparison of AneuFinder copy number calling of T257; comparing the fit when forcing AneuFinder to call the majority state tetrasomy (*left*) or disomy (*right*). (PDF 4660 kb)

Additional file 16: Figure S9. Additional scWGS data for human B-ALLs. **a** Copy number plots showing the reads per 1 Mb of cumulative single-cell sequencing data analysed as simulated bulk, showing an obscuring effect on the karyotype heterogeneity. **b** Genomic context of the CNV on chromosome 2 in B-ALL B. (PDF 1964 kb)

Additional file 17: Figure S10. Chimerism levels for B-ALL B over time. Chimerism is defined as >1 % hCD45+ peripheral blood mononuclear cells (PBMCs). Plotted is the percentage of hCD45+ PBMCs at 4-week intervals B-ALL B (*n* = 2). Mice engrafted with B-ALL B showed ~80 % chimerism after 28 weeks at which time they were sacrificed. (PDF 324 kb)
