# Supplementary figures and images for: Single-cell sequencing reveals karyotype heterogeneity in murine and human malignancies
Source: Genome Biol. 2016 May 31;17:115. doi: 10.1186/s13059-016-0971-7 (PMC4888588; doi:10.1186/s13059-016-0971-7)

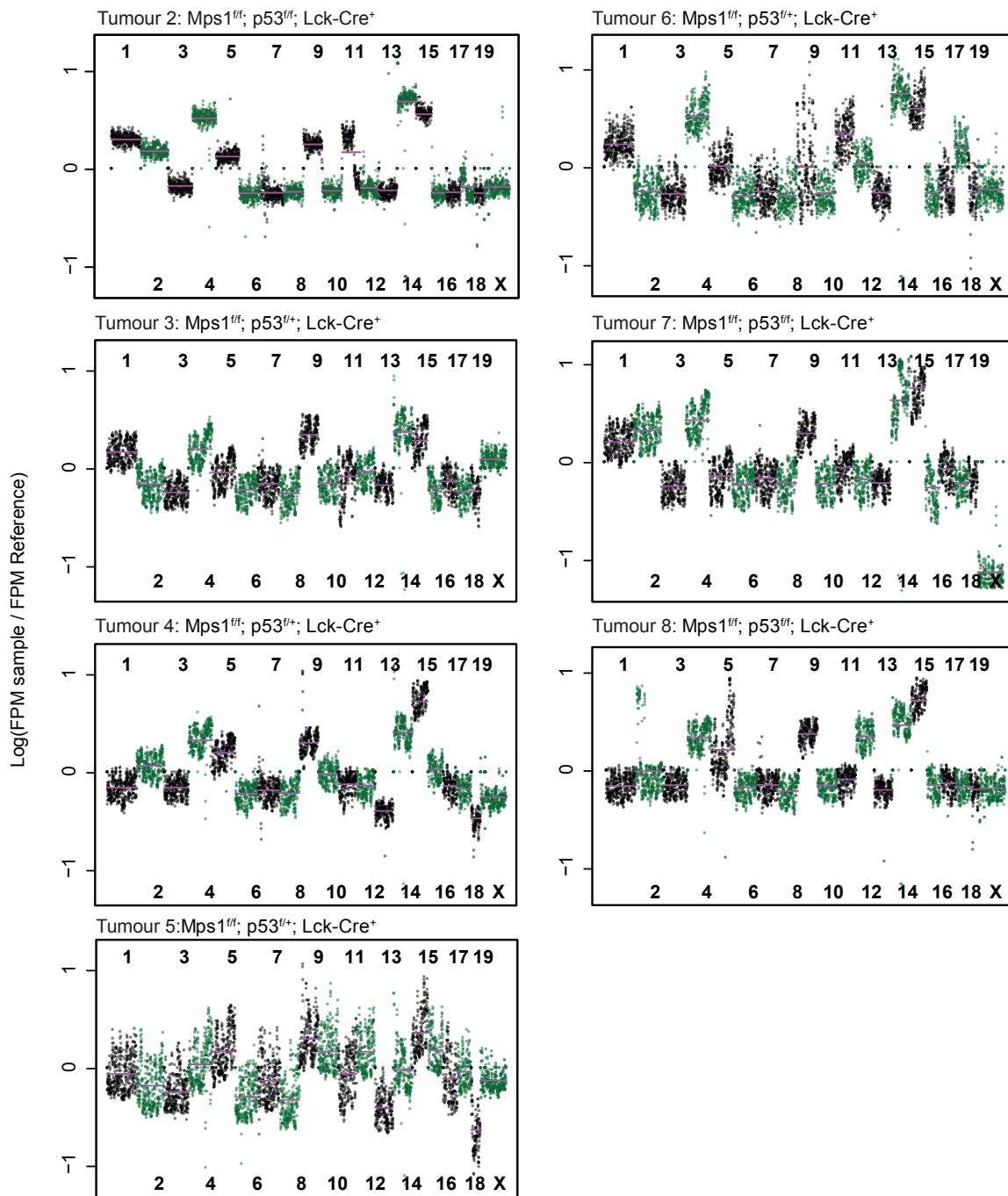

Supplement: Supplementary file 1 — Additional comparisons of aCGH and exome-sequencing analyses of T-ALLs driven by Mps1 and p53 mutation. Six additional T-ALLs analysed using array CGH, compared to a euploid reference, showing recurrent gains of predominantly chromosomes 4, 9, 14 and 15, and other lymphoma-specific alterations. (PDF 23139 kb) [file 13059_2016_971_MOESM1_ESM.pdf]

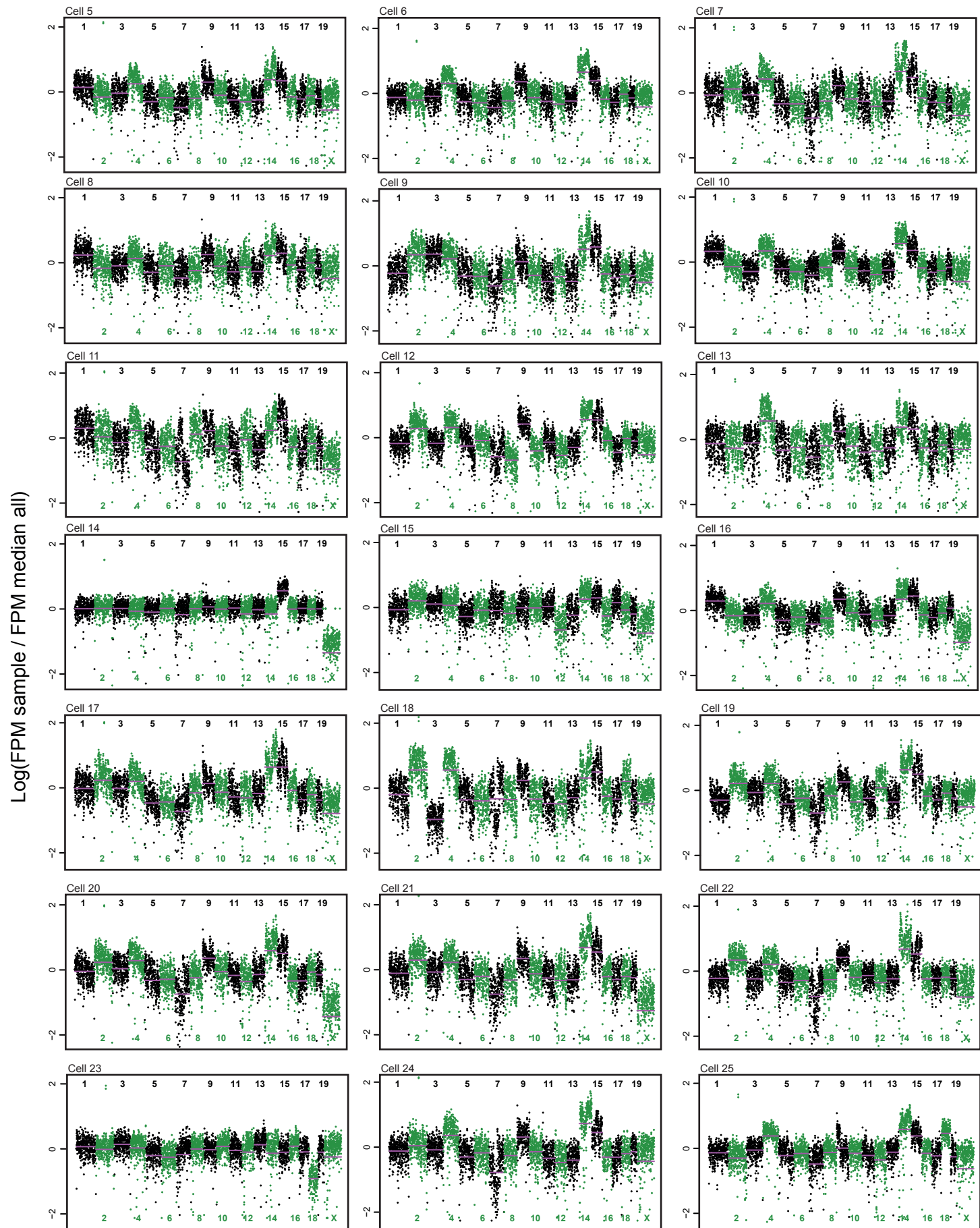

Supplement: Supplementary file 2 — Additional single-cell sequencing analyses of Mps1 T-ALL 1. Single-cell sequencing plots for Mps1 T-ALL cells 1 (continuation of Fig. 1c). (PDF 63616 kb) [file 13059_2016_971_MOESM2_ESM.pdf]

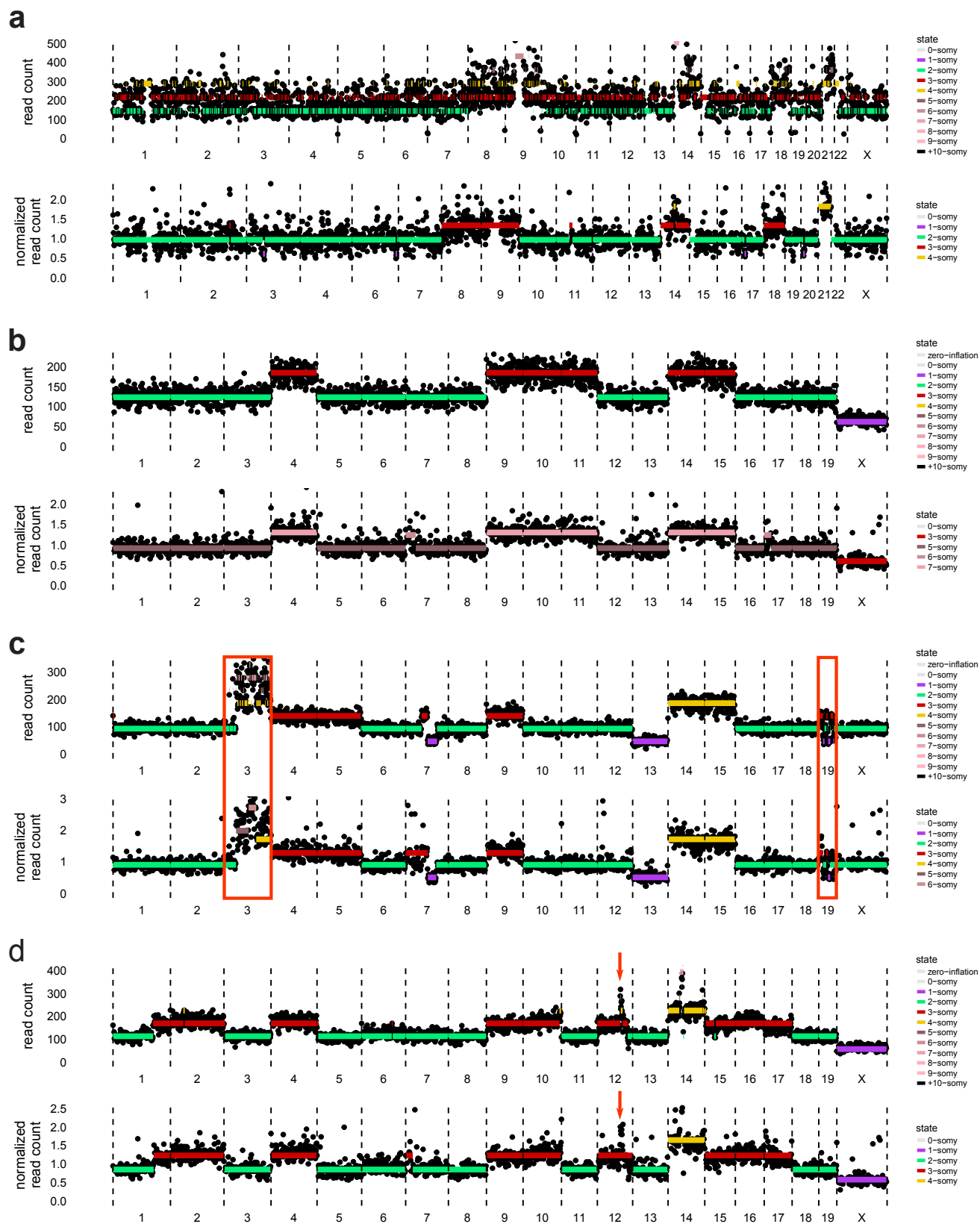

Supplement: Supplementary file 7 — Examples of discordant copy number calls between AneuFinder and Ginkgo. Top panels show the AneuFinder profiles, bottom panels show the Ginkgo profiles, respectively. a Low quality library showing a highly segmented fit with AneuFinder. b Wrongly chosen ploidy state with Ginkgo. c Red boxes indicate chromosomes with unusually high read count dispersion where AneuFinder fails to assign a clear copy number state. d Small copy number change that is detected with AneuFinder but not with Ginkgo. (PDF 2236 kb) [file 13059_2016_971_MOESM7_ESM.pdf]

# Cumulative single-cell seq libraries: T-ALLs

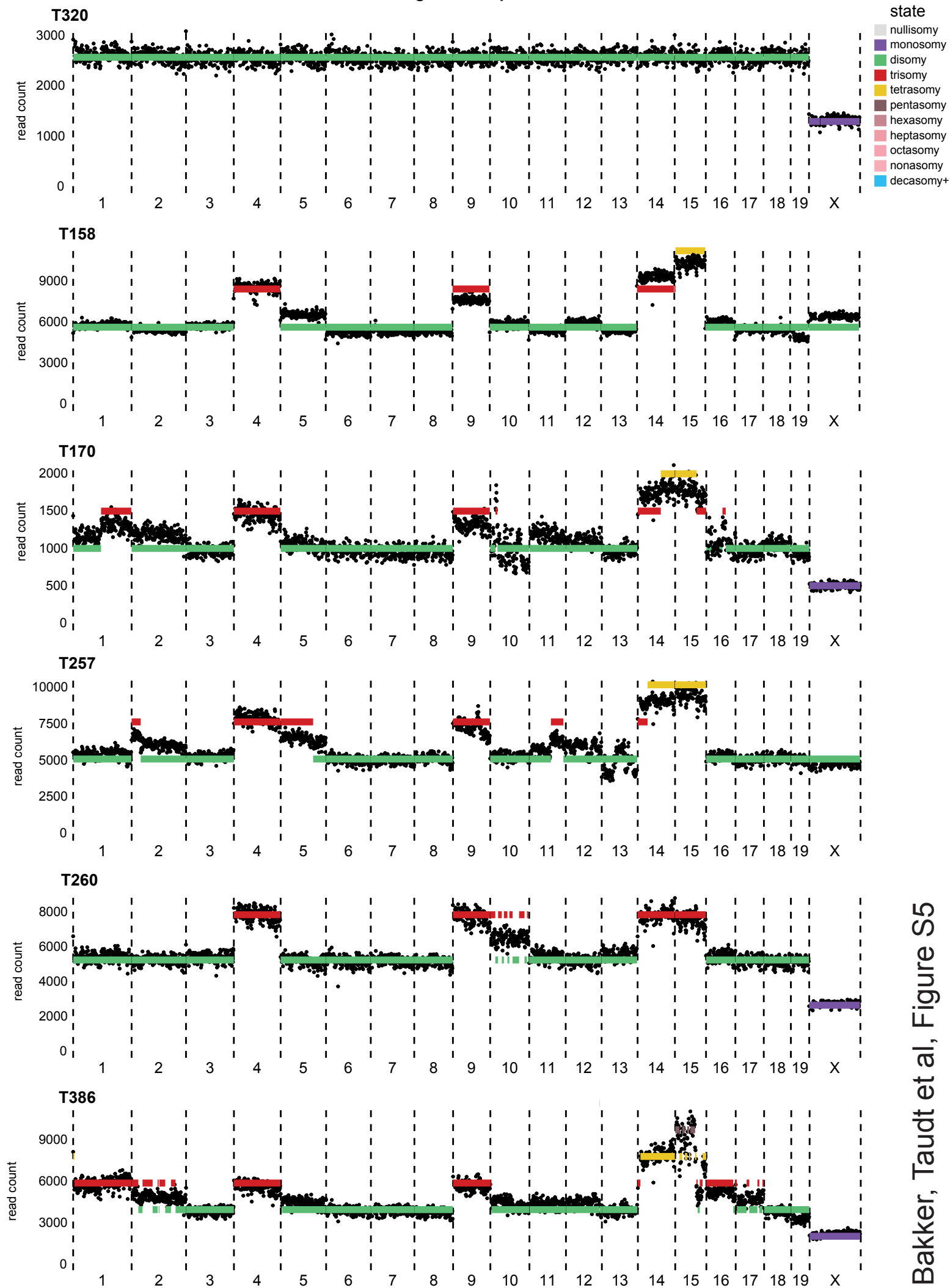

Supplement: Supplementary file 8 — Cumulative single-cell sequencing data of control thymus and aneuploid T-ALLs. Copy number plots showing the reads per 1 Mb of cumulative single-cell sequencing data analysed as simulated bulk data, showing an obscuring effect on the karyotype heterogeneity. (PDF 3267 kb) [file 13059_2016_971_MOESM8_ESM.pdf]

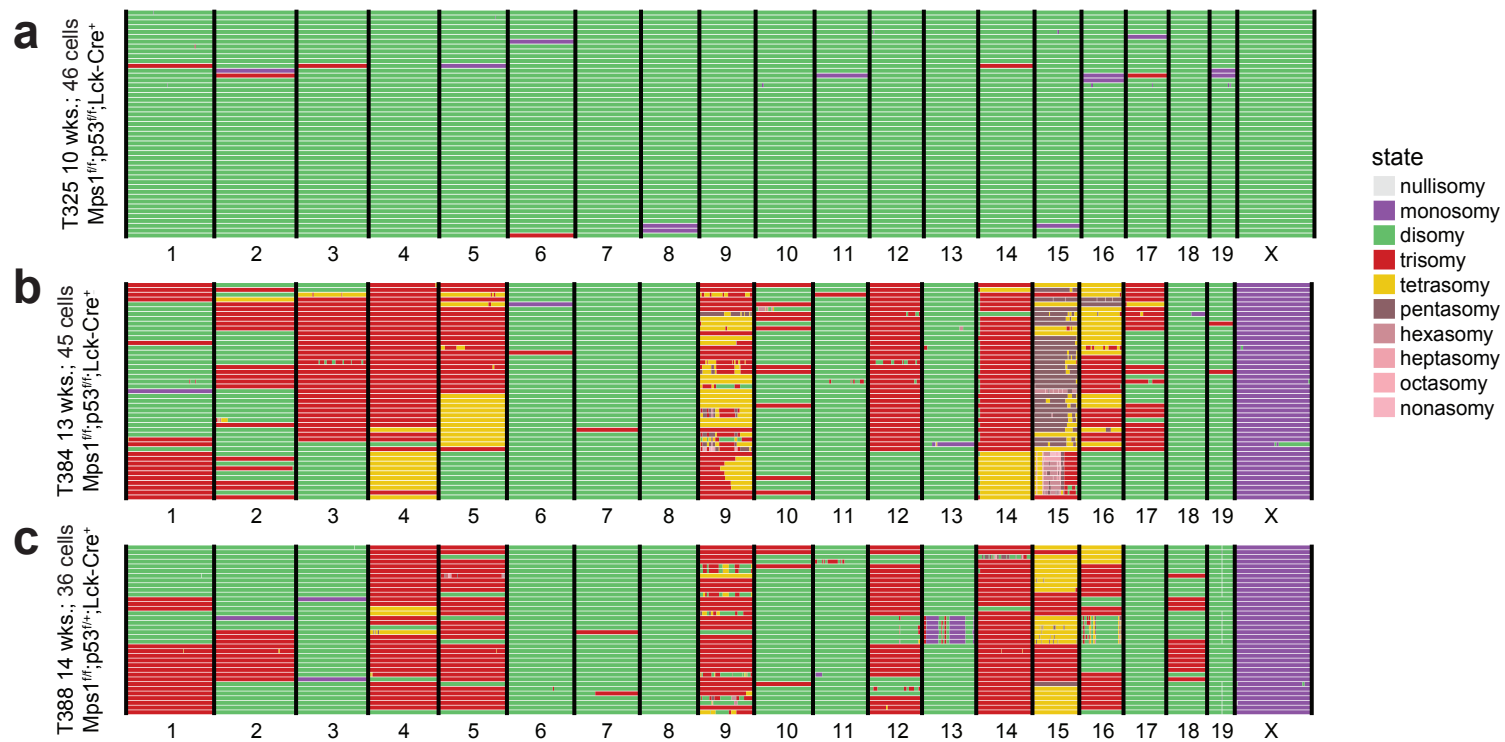

Bakker, Taudt et al, Figure S6

Supplement: Supplementary file 9 — Single-cell sequencing of early time point T-ALLs. Genome-wide copy number plots using ~1 Mb bins for three thymuses harvested from 10-, 13- and 14-week-old mice, showing high levels of karyotype heterogeneity at 13 and 14 weeks. (PDF 451 kb) [file 13059_2016_971_MOESM9_ESM.pdf]

# Aneuploidy and heterogeneity scores per chromosome

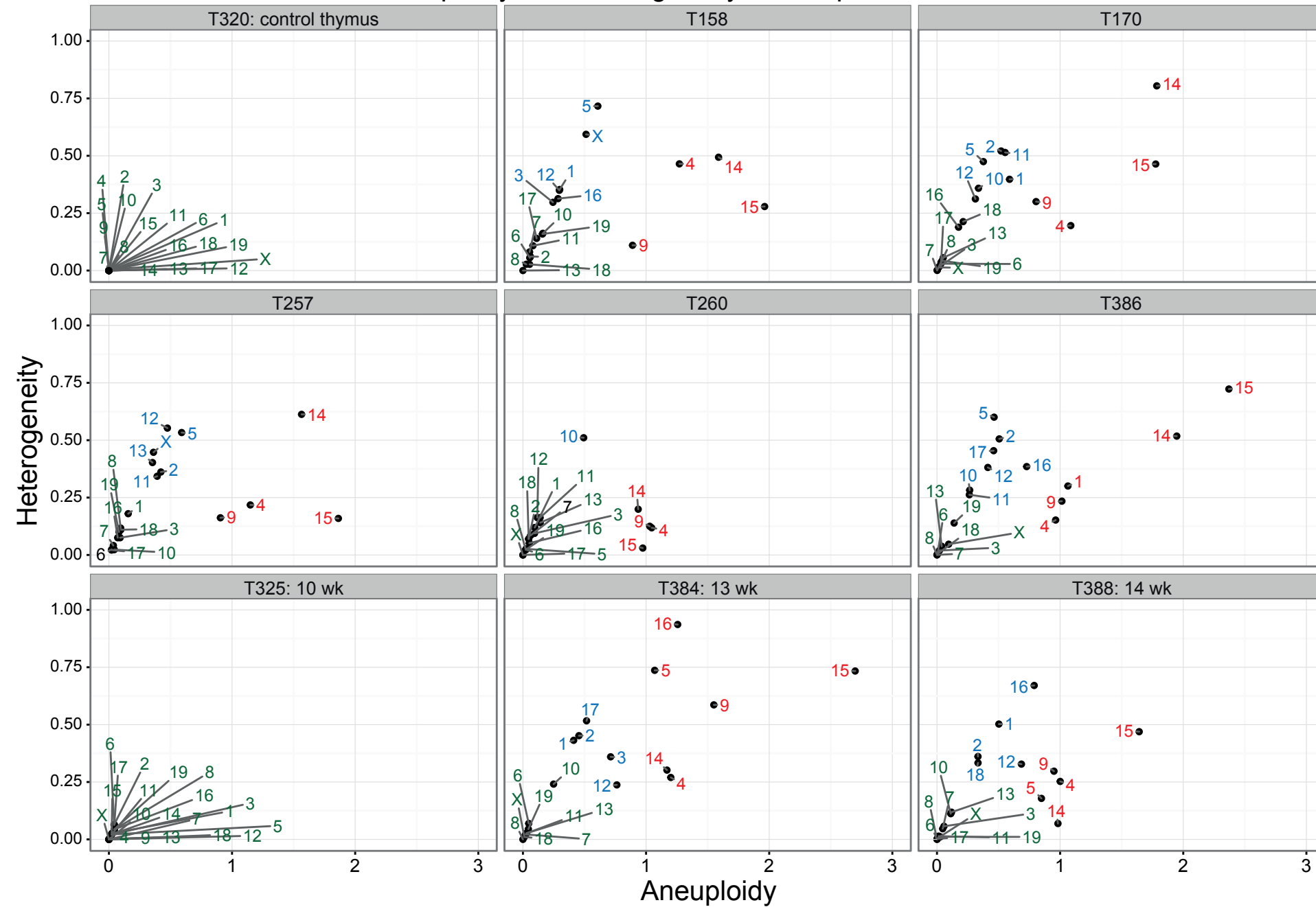

Supplement: Supplementary file 10 — Aneuploidy and heterogeneity per chromosome observed in a control thymus and T-ALLs. Aneuploidy and heterogeneity scores plotted per chromosomes of all T-ALLs examined in the study. Chromosomes indicated in green do not favour copy number change and show minimal heterogeneity. Chromosomes in blue show apparent random copy number changes. Red chromosomes favour copy number changes. (PDF 440 kb) [file 13059_2016_971_MOESM10_ESM.pdf]

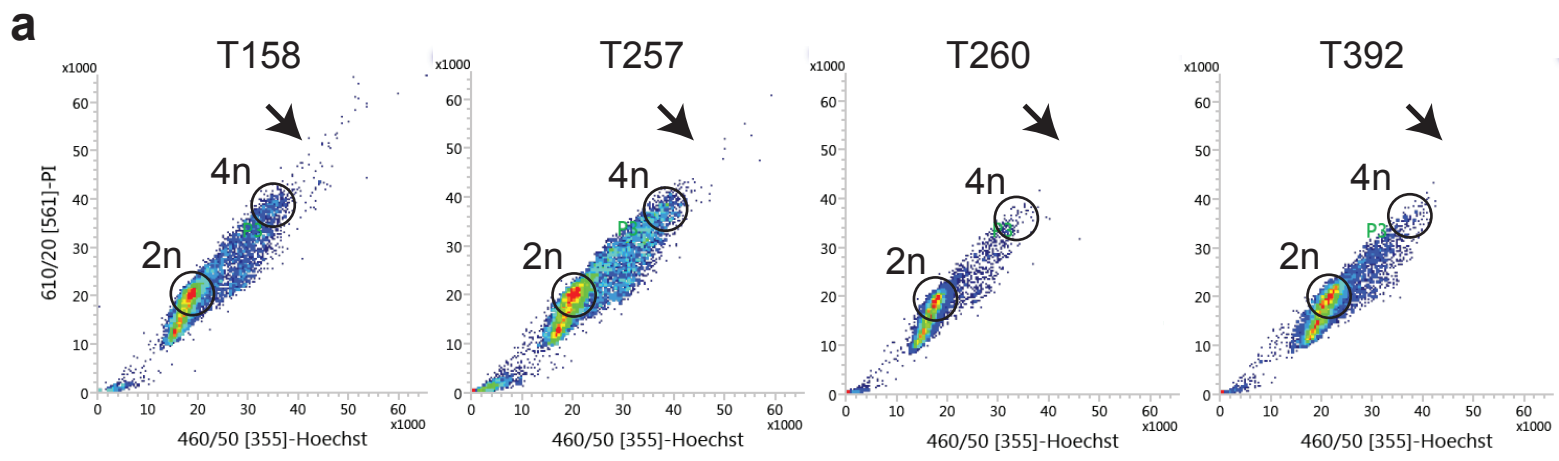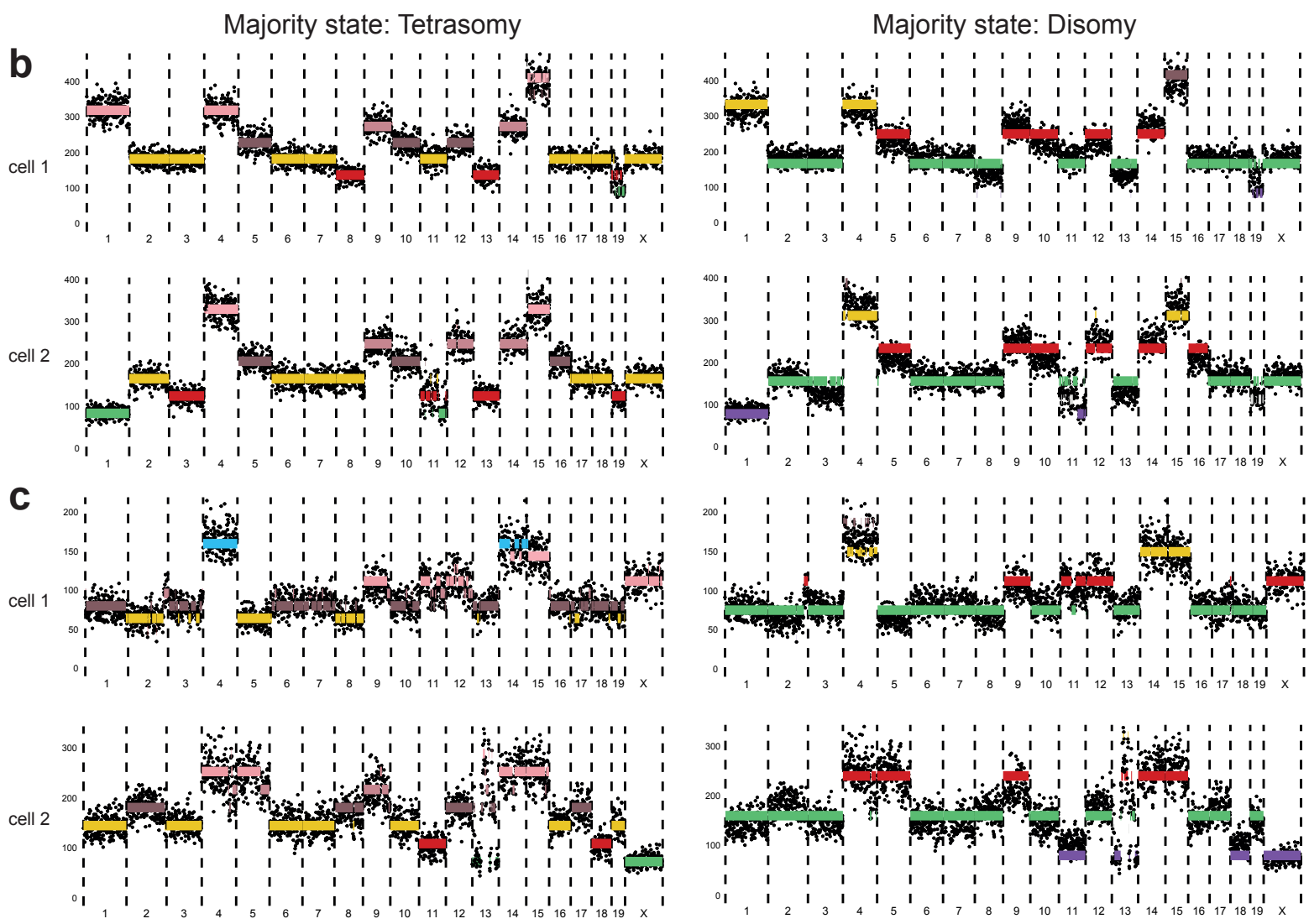

Supplement: Supplementary file 15 — Single-cell sequencing of (near)-4n cells in T158 and T257. a PI/Hoechst FACS plots showing for four tumours, showing apparent cycling tetraploid cells in T158 and T257. b Comparison of AneuFinder copy number calling of T158; comparing the fit when forcing AneuFinder to call the majority state tetrasomy (left) or disomy (right). c Comparison of AneuFinder copy number calling of T257; comparing the fit when forcing AneuFinder to call the majority state tetrasomy (left) or disomy (right). (PDF 4660 kb) [file 13059_2016_971_MOESM15_ESM.pdf]

# Cumulative single-cell seq libraries: B-ALLs

**a**

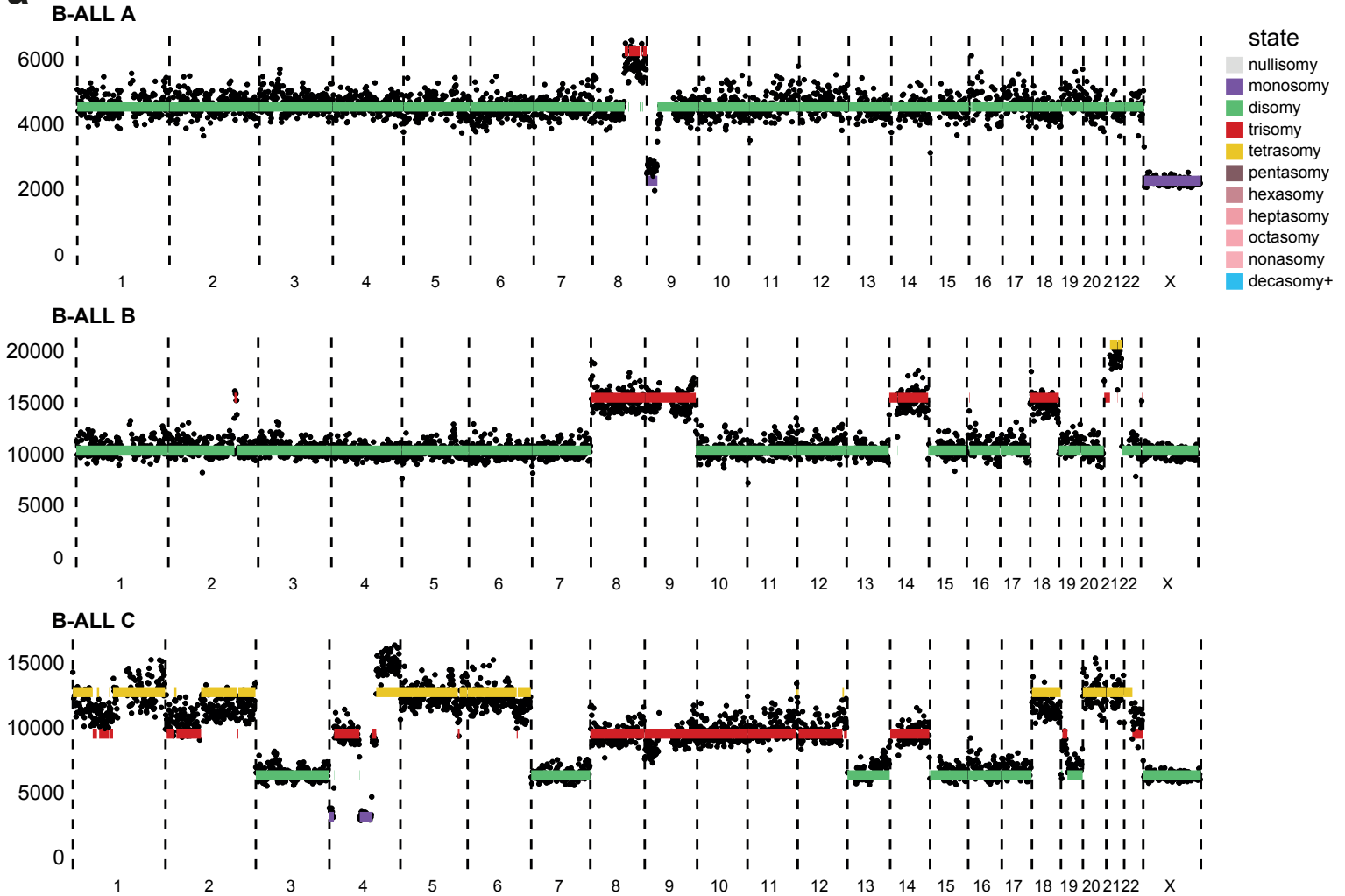

**b**

GRCh37/hg19 - chr2:177,992,747-188,321,146

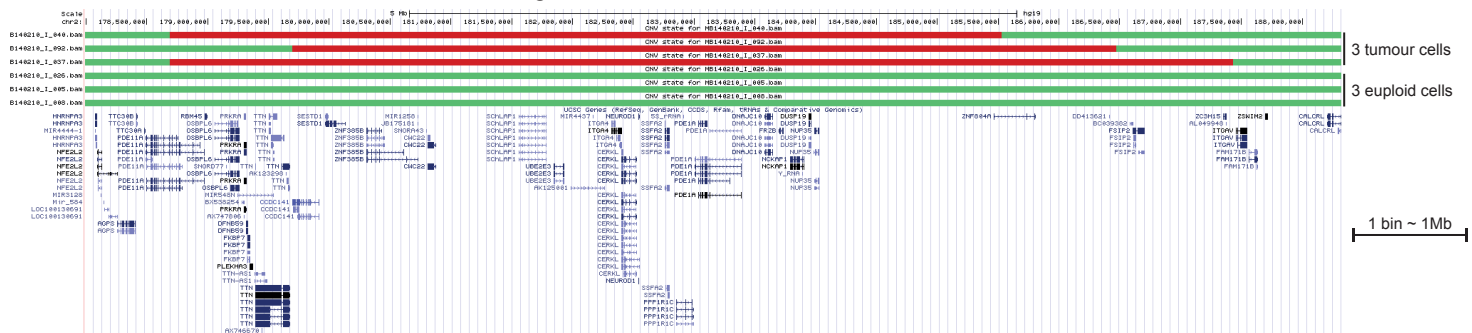

Bakker, Taudt et al, Figure S9

Supplement: Supplementary file 16 — Additional scWGS data for human B-ALLs. a Copy number plots showing the reads per 1 Mb of cumulative single-cell sequencing data analysed as simulated bulk, showing an obscuring effect on the karyotype heterogeneity. b Genomic context of the CNV on chromosome 2 in B-ALL B. (PDF 1964 kb) [file 13059_2016_971_MOESM16_ESM.pdf]

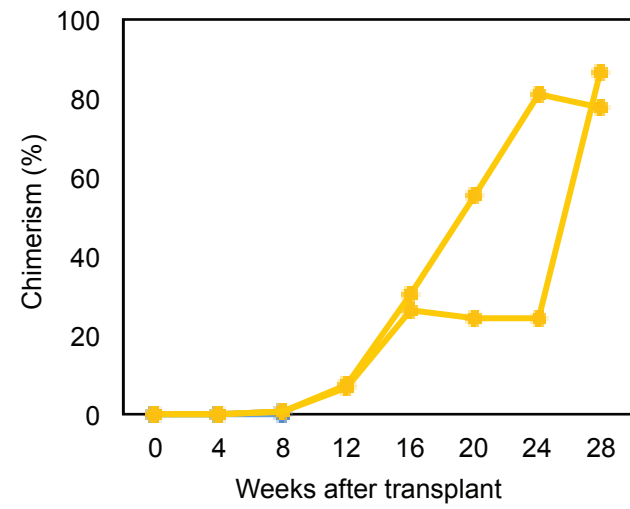

Bakker, Taudt et al, Figure S10

Supplement: Supplementary file 17 — Chimerism levels for B-ALL B over time. Chimerism is defined as >1 % hCD45+ peripheral blood mononuclear cells (PBMCs). Plotted is the percentage of hCD45+ PBMCs at 4-week intervals B-ALL B (n = 2). Mice engrafted with B-ALL B showed ~80 % chimerism after 28 weeks at which time they were sacrificed. (PDF 324 kb) [file 13059_2016_971_MOESM17_ESM.pdf]
