# Supplementary material for: Single-cell sequencing reveals karyotype heterogeneity in murine and human malignancies
Source: Genome Biol. 2016 May 31;17:115. doi: 10.1186/s13059-016-0971-7 (PMC4888588; doi:10.1186/s13059-016-0971-7)
Supplement: Supplementary file 3 — Quantification of single-cell sequencing data of Mps1 T-ALL 1. a Quantification of the gains and losses of chromosomes for 25 Mps1 T-ALL 1 cells as analysed using single-cell sequencing (Additional file 2: Figure S2). Losses and gains were scored as 1 s and 3 s, respectively; 2 s indicates no change or disomies. The focal loss on chromosomes 7 was scored as a loss to discriminate cells that did not show this CNV. Fourteen out of 25 cells (56 %) displayed a unique karyotype. Cells with identical karyotypes are clustered together, resulting in 18 groups. b Frequency percentages of the gain, no change and loss events for chromosomes of Mps1 T-ALL 1. Gain of chromosome 4, 9, 14 and 15 are the most frequent events, occurring in >90 % of the cells, with gain of chromosomes 2 and the focal loss on chromosome 7 occurring in ~50 % of the cells. (PDF 548 kb) [file 13059_2016_971_MOESM3_ESM.pdf]

a

Cell ID	1	2	3	4	5	6	7	8	9	10	11	12	13	14	15	16	17	18	19	X	Group ID
23	2	2	2	2	2	2	2	2	2	2	2	2	2	2	2	2	2	1	2	2	1
14	2	2	2	2	2	2	2	2	2	2	2	2	2	2	3	2	2	2	2	1	2
25	2	2	2	3	2	2	2	2	2	2	2	2	2	3	3	2	2	3	2	2	3
6	2	2	2	3	2	2	2	2	3	2	2	2	2	3	3	2	2	2	2	2	4
13	2	2	2	3	2	2	2	2	3	2	2	2	2	3	3	2	2	2	2	2	
18	2	3	1	3	2	2	2	2	3	2	2	2	2	3	3	2	2	3	2	2	5
12	2	3	2	3	2	2	1	1	3	2	3	2	2	3	3	2	2	2	2	2	6
1	2	3	2	3	2	2	1	2	3	2	2	2	2	3	3	2	2	2	2	1	7
20	2	3	2	3	2	2	1	2	3	2	2	2	2	3	3	2	2	2	2	1	
21	2	3	2	3	2	2	1	2	3	2	2	2	2	3	3	2	2	2	2	1	
7	2	3	2	3	2	2	1	2	3	2	2	2	2	3	3	2	2	2	2	2	8
22	2	3	2	3	2	2	1	2	3	2	2	2	2	3	3	2	2	2	2	2	
24	2	3	2	3	2	2	1	2	3	2	2	2	2	3	3	2	2	2	2	2	
19	2	3	2	3	2	2	1	2	3	2	2	3	2	3	3	2	2	2	2	2	9
17	2	3	2	3	2	2	2	2	3	2	2	2	2	3	3	2	2	2	2	2	10
9	2	3	3	3	2	2	1	2	3	2	2	2	2	3	3	2	2	2	2	2	11
2	2	3	3	3	2	2	1	2	3	2	3	2	2	3	3	2	1	2	2	1	12
15	2	3	3	3	2	2	2	2	2	2	2	1	2	3	3	2	3	2	2	1	13
4	3	2	1	3	2	2	2	1	3	3	1	2	1	3	2	2	2	2	1	2	14
16	3	2	2	3	2	2	2	2	3	2	2	2	2	3	3	2	2	2	2	1	15
5	3	2	2	3	2	2	2	2	3	2	2	2	2	3	3	2	2	2	2	2	16
8	3	2	2	3	2	2	2	2	3	2	2	2	2	3	3	2	2	2	2	2	
10	3	2	2	3	2	2	2	2	3	2	2	2	2	3	3	2	2	2	2	2	
3	3	2	2	3	2	2	2	2	3	2	2	2	2	3	3	2	2	2	2	3	17
11	3	3	2	3	2	2	1	3	3	2	2	2	2	3	3	2	2	2	2	1	18

Cells with a unique karyotype:
14 / 25 (56%)

Groups of cells with identical karyotypes:
18

b

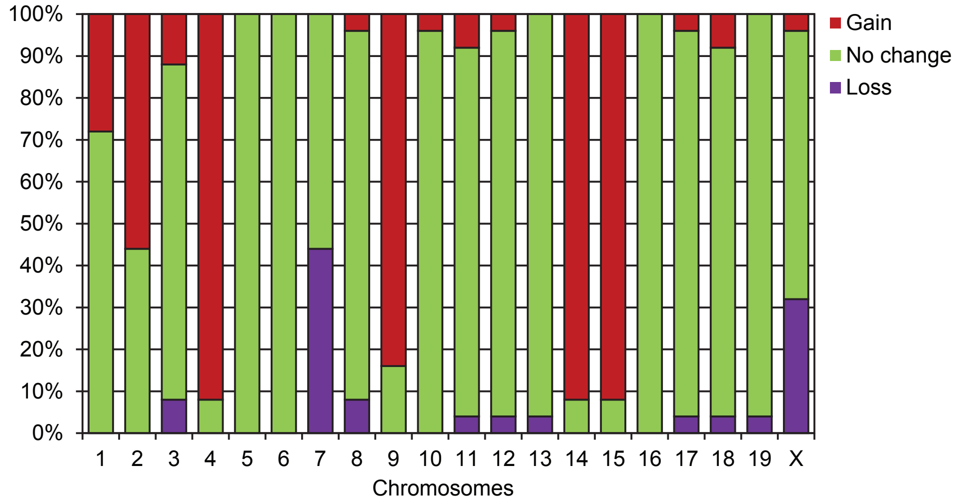

Bakker, Taudt et al, Figure S3
