# Supplementary material for: Single-cell sequencing reveals karyotype heterogeneity in murine and human malignancies
Source: Genome Biol. 2016 May 31;17:115. doi: 10.1186/s13059-016-0971-7 (PMC4888588; doi:10.1186/s13059-016-0971-7)
Supplement: Supplementary file 5 — Supplementary Materials and Methods. (DOCX 35 kb) [file 13059_2016_971_MOESM5_ESM.docx]

**Additional file 5: Supplementary Materials and Methods**

For each single cell, copy-number detection using AneuFinder consists of three steps: (1) Binning, (2) correction for GC content, and (3) copy-number detection with a Hidden Markov Model. This is followed by a semi-automated quality control for all libraries using a multivariate clustering approach [1, 2].

*Binning*

We implemented two different binning strategies, fixed-width and variable-width windows. In the fixed-width binning strategy, we partition the genome into *T* non-overlapping, equally sized bins (default 1Mb) and count the number of aligned reads that overlay any given bin *t*. The variable-width binning requires a euploid reference that can either be a simulated or be a real reference (*e.g.* many merged euploid single cell libraries). The bins are constructed as follows: 1) The euploid reference is binned into fixed-width windows of a given size (default 1Mb) and reads are counted in each bin. 2) The mode of read counts per fixed-width bin (*X)* is taken as the desired number of reads for the variable-width bins. 3) Variable-width bins are constructed such that each bins contains *X* reference reads. Fixed-width windows can lead to artefacts in the form of low copy number states that are caused by low-mappability regions. We therefore conducted all analyses in this manuscript with the variable-width binning approach, which partly corrects for mappability bias. Reference files for this study were generated by merging reads from 46 diploid single cells for mouse (thymus T320) and 52 diploid single cells for human (reference to van den Bos *et al*, page XXX of this issue).

*Blacklist*

Variable-width bins offer a partial correction for mappability, however, even with variable-width bins we could still observe artefacts around centromeric regions. We chose a blacklisting strategy to exclude reads from artefact-prone regions from the analysis. Blacklists for this study were generated by binning the references into fixed-width bins of 100 kb and blacklisting all bins where the read count was above the 0.9985 quantile or below the 0.1 quantile, respectively.

*GC correction*

We partition the genome into *T* non-overlapping, variable or fixed-width bins as described above and count the number of aligned reads that fall into any given bin *t* *[3]*. This read count *x_t_* is further GC-corrected with a model modified from [4]. For every bin *t* the GC content is determined as a fraction between 0 and 1, and the read count *x_t_* multiplied by a correction factor *f_GC_* that is dependent on the GC content.

$x_{t,corrected}=x_{t}\cdot f_{GC}$ (1)

To calculate the correction factor, we group bins with GC content in one of 20 equally spaced intervals between 0 to 1 and calculate a correction factor *f’_GC_* as follows

${f'}_{GC}=\frac{x_{global}}{x_{GC}}$ (2)

where $x_{global}$ is the average read count over all bins and $x_{GC}$ is the average read count for all windows with a GC content in one of 20 equally spaced intervals between 0 to 1 (we use a trimmed mean, omitting 5% of bins from both extremes). Finally, we obtain the correction factor $f_{GC}$ by fitting a second order polynomial to the correction factor ${f'}_{GC}$. Corrected read counts are rounded to the nearest integer.

*Hidden Markov Model*

Each copy number is modelled by a distinct hidden state in our Hidden Markov Model (HMM). The read count distribution for each copy number ≥ 1 is modelled by a negative binomial distribution:

$NB\left( r,p,x_{t} \right)=\frac{\Gamma\left( x_{t}+r \right)}{\Gamma\left( r \right)x_{t}!}p^{r}\left( 1-p \right)^{x_{t}}$ (3)

The probability parameter *p* is the same for all states ≥ 1 (monosomy, disomy, trisomy, etc.) and the dispersion parameters *r* are multiples of the dispersion parameters for state 1 (monosomy) (see Figure 2c for a graphical representation). Nullisomies (copy number 0) are modelled by two hidden states: One “zero-inflation” state with a delta distribution to model gaps where no reads can be aligned and one state with a geometric distribution to account for mis-mapping reads even in the case of zero copies:

$Geom\left( p,x_{t} \right)=p\left( 1-p \right)^{x_{t}}$ (4)

We use the Baum-Welch algorithm [5] to obtain a fit for the distribution parameters, transition probabilities and posterior probabilities. For each bin, we assign the copy number state as the state with highest posterior probability. The number of copies that are modelled by our HMM can be specified by the user (default 10).

*Quality control*

Quality control has to be an integral part of the analysis pipeline, since single-cell sequencing libraries can be inherently noisy. Furthermore, a simple cut-off on total read coverage is often not informative because even libraries with many reads can have a very non-uniform coverage. We have developed several measures to assess library quality:

- The **spikiness** *s* of a library is a measure for the bin-to-bin variation of the read count $x_{t}$ and is defined as:

$s=\frac{\sum_{t=1}^{T-1} \left| x_{t+1}-x_{t} \right|}{\sum_{t=1}^{T} x_{t}}$ (4)

- By contrast, the **shannon entropy** *e* for the read count is a measure of the uniformity of the read distribution and is defined as:

$e=\frac{-\sum_{t=1}^{T} x_{t}}{X\cdot log\left( \frac{x_{t}}{X} \right)}$ (5)

where *X* is the sum over all read counts: $X=\sum_{t^{'}=1}^{T} x_{t'}$.

- We found that the **loglikelihood** of the model as determined from the Baum-Welch algorithm is also a good measure to discriminate libraries by quality.
- The **number of copy number segments** can also be used to assess library quality. A segment is defined as a continuous stretch of bins with the same copy number state. This number will be high for bad quality libraries and low for good quality libraries.
- The **Bhattacharyya distance** *b* is a measure of how well two distributions can be distinguished and is defined as:

$b=-log\left[ \sum_{x} \sqrt{{NB}_{1}\left( r,p,x_{t} \right)*{NB}_{2}\left( r,p,x_{t} \right)} \right]$ (6)

where NB_1_ is the negative bionomial distribution for the state monosomy and NB_2_ is the negative bionomial distribution for the state disomy, and *r* and *p* are the dispersion and probability parameters thereof, respectively.

Please note that spikiness and shannon entropy are defined on the read count, while loglikelihood, segment number, and Bhattacharyya distance are defined on the output of the Hidden Markov Model. While all of those measures allow quality assessment of single cell libraries, we found that none of those measure alone was powerful enough to reliably discriminate good quality and bad quality libraries. Therefore, we employed a multivariate clustering approach implemented in the R-package mclust [1, 2] to utilize all quality criteria simultaneously to discriminate libraries by quality.

*Karyotype measures*

To assess karyotype heterogeneity and the level of aneuploidy in populations of single cells we developed two measures that aggregate information over the population of single cells. For a set of *N* single cells with *T* bins, we define an aneuploidy score as:

$\begin{aligned} \\ D=\frac{1}{TN}\sum_{n=1}^{N} \sum_{t=1}^{T} |c_{n,t}-e_{t}| \end{aligned}$ (7)

where *c_n,t_* is the copy number state of cell *n* at bin *t*, and *e_t_* is the euploid copy number at bin *t* (*e.g.* *e =* 2 for autosomes, and *e* = 2 or 1 for the female or male X-chromosome respectively).

We define a heterogeneity score as:

$H=\frac{1}{TN}\sum_{t=1}^{T} \sum_{f=0}^{S} f\cdot m_{f,t}$ (8)

where *m_f,t_* is the number of cells with copy number state *s* at bin *t*, and *S* is the total number of copy number states. Now, importantly, the *m_f,t_* is ordered for each bin such that *m_f_*_=0,t_ ≥ *m_f_*_=1,t_ ≥ *m_f_*_=2,t_, etc. in such a way that *f* is not necessarily equal to *s.* Each type of aneuploidy (e.g. monosomy, trisomomy, tetrasomy etc.) has an equal impact on this score, further evidenced by simulating various aneuploid conditions and calculating the aneuploidy and heterogeneity scores (Table S2).

*Implementation*

The software was implemented as R-package *AneuFinder* and is freely available from <https://github.com/ataudt/aneufinder>. It integrates with the popular Bioconductor environment to facilitate downstream bioinformatics analysis. A tutorial for AneuFinder that will be updated regularly can be found at <http://aneuploidy.nl/aneufinder>.

*Comparison to other methods*

We compared AneuFinder with a web based tool for single cell CNV calling based on Circular Binary Segmentation (Ginkgo [6]). We analysed 325 cells from tumours T158, T170, T257, T260, T386 and B-ALL-B with Ginkgo (1Mb variable-width bins based on simulated reads of 48bp mapped with bowtie, otherwise default parameters) and AneuFinder at variable bin size 1Mb, and found that 81% (263/325) of the cells had concordant copy number calls for more than 95% of base pairs. Of the remaining 19% (62/325) of discordant cells, approximately half (27/62) were due to failed libraries. The other half (35/62) was caused by incorrect fits due to sequencing noise for AneuFinder (Fig. S4a) or wrongly chosen ploidy state for Ginkgo (Fig. S4b). However, the AneuFinder pipeline would filter out these problematic fits (like in Fig. S4a) in the quality control step and therefore these cells would not be used for subsequent analysis.

While Ginkgo was more robust to sequencing noise (Fig. S4a,c), we found it to be less sensitive for the detection of small CNVs of only a couple of bins (Fig. S4d). Another important advantage of AneuFinder is that it is available as an R-package, offering more flexibility for the analysis of non-standard genomes and sequencing parameters.

**Supplementary references**

1. Fraley C, Raftery A: **Model-based Clustering, Discriminant Analysis and Density Estimation**. *J Am Stat Assoc* 2002, **97**:611–631.

2. Fraley C, Raftery A, Murphy T, Scrucca L: *Mclust Version 4 for R: Normal Mixture Modeling for Model-Based Clustering, Classification, and Density Estimation*. 2012.

3. Lawrence M, Huber W, Pagès H, Aboyoun P, Carlson M, Gentleman R, Morgan MT, Carey VJ: **Software for computing and annotating genomic ranges.** *PLoS Comput Biol* 2013, **9**:e1003118.

4. Teo SM, Pawitan Y, Ku CS, Chia KS, Salim A: **Statistical challenges associated with detecting copy number variations with next-generation sequencing**. *Bioinformatics* 2012, **28**:2711–2718.

5. Baum L, Petrie T, Soules G, Weiss N: **A Maximization Technique Occurring in the Statistical Analysis of Probabilistic Functions of Markov Chains**. *Ann Math Stat* 1970, **41**:164–171.

6. Garvin T, Aboukhalil R, Kendall J, Baslan T, Atwal GS, Hicks J, Wigler M, Schatz MC: **Interactive analysis and assessment of single-cell copy-number variations**. *Nat Methods* 2015, **12**:1058–1060.
